# Silicon and methionine enhance cowpea water stress tolerance

**DOI:** 10.1038/s41598-026-37795-2

**Published:** 2026-01-31

**Authors:** Guilherme Félix Dias, Semako Ibrahim Bonou, Priscylla Marques de Oliveira Viana, Rayanne Silva de Alencar, Túlio William da Silva Gonçalves, Igor Eneas Cavalcante, Agda Malany Forte de Oliveira, Railene Hérica Carlos Rocha Araújo, Maurisrael de Moura Rocha, Alberto Soares de Melo

**Affiliations:** 1 Programa de Pós-Graduação Em Ciências Agrárias, Universidade Estadual da Paraíba, Campina Grande, PB 58429-500 Brazil; 2 Departamento de Engenharia Agrícola, Universidade Federal de Campina Grande, Campina Grande, PB 58429-900 Brazil; 3 Departamento de Biologia, Universidade Estadual da Paraíba, Campina Grande, PB 58429-500 Brazil; 4 Departamento de Ciências Vegetais E Ambientais, Universidade Federal da Paraíba, Areia, PB 58051-900 Brazil; 5 Centro de Tecnologia de Recursos Naturais, Universidade Federal de Campina Grande, Campina Grande, PB 58429-900 Brazil; 6 Embrapa Meio-Norte, Teresina, PI 64008-780 Brazil

**Keywords:** *Vigna unguiculata* (L.) Walp., Water stress, Silicic acid, L-methionine, Physiological plasticity, Physiology, Plant sciences

## Abstract

This study investigated whether foliar application of silicon (Si) and methionine (Met) can modulate the biochemical metabolism and growth of cowpea ‘BRS Exuberante’ during water restriction and rehydration cycles, based on the hypothesis that these elicitors could improve physiological recovery after water stress. Considering the above, the experiment was conducted in a completely randomized design, in a 2 × 4 factorial scheme, with four replications. The factors studied were: water stress period and rehydration period. In addition, the following foliar doses of the elicitors were applied: control, 300 mg L-1 of Si, 890 mg L-1 of Met, and the combination of both, 300 mg L-1 of Si + 890 mg L-1 of Met. The results that positively impacted the mitigation of water stress observed in the presence of silicon were more related to growth, although they also reflected improvements in biochemical metabolism. Methionine, on the other hand, was associated with changes in biochemical aspects that benefited growth. In both cases, the improvements that occurred during water restriction were what enabled greater recovery potential after rehydration. Both attenuating agents proved effective in inducing tolerance to certain physiological characteristics. Therefore, their use represents a promising alternative to strengthen the tolerance mechanisms of cowpea, especially by stimulating its ability to recover from stress after rehydration.

**Supplementary Information:**

The online version contains supplementary material available at 10.1038/s41598-026-37795-2.

## Introduction

Long periods of drought, caused primarily by irregular rainfall and intensified by climate change, have become more frequent and constitute one of the main and most severe abiotic stressors affecting plant physiology, especially in arid and semiarid regions. It is noteworthy that plants have defense mechanisms that, in addition to enabling survival during drought, promote more efficient recovery after the return of adequate water conditions, thus representing a key aspect of plant resilience to drought^1,2^. This phenomenon is particularly evident in perennial species, whose post-drought recovery responses have been extensively documented in the literature^3–5^. However, there are still gaps in knowledge regarding post-drought dynamics in annual species, especially cowpea [*Vigna unguiculata* (L.) Walp.], considered a strategic crop for semiarid regions^6^.

In general, the physiological mechanisms involved in drought tolerance in cowpea include maintaining plant water status through osmotic adjustment, with the accumulation of organic solutes such as proline and soluble sugars; controlling stomatal conductance; increasing the activity of antioxidant enzymes; and changes in cells, organs, and the structure of the plant as a whole. These adaptive mechanisms enable plants to conserve water for use in subsequent periods, thus increasing water resource efficiency^7,8^. In this context, it is essential to deepen our understanding of the physiological mechanisms related to drought resistance and recovery from water stress, considering the frequency with which such conditions occur in agricultural crops. In addition to understanding these processes, it is necessary to identify strategies that enhance recovery by strengthening plants’ natural defenses, particularly through the foliar application of elicitors such as silicon and methionine^9,10^. These attenuators have been described in the literature as beneficial to plants under stress conditions.

Silicon, for example, is effective in inducing tolerance, acting to enhance antioxidant mechanisms, regulate osmotic balance, reduce the production of reactive oxygen species (ROS), and maintain cell turgor. It also strengthens the cell wall and membrane, mitigating damage caused by water deficit^11,12^. In turn, methionine is an amino acid that can act as a coenzyme and precursor to several bioregulators and signaling molecules, such as jasmonic acid and salicylic acid, promoting systemic acquired resistance, improving photosynthesis, and regulating processes associated with ethylene biosynthesis and sulfur metabolism. In this sense, methionine also plays a crucial role in plant growth and development^13–15^. However, information regarding its effects on the recovery process after periods of water restriction is still insufficient.

Given the aforementioned information, studies on the recovery capacity of annual plants, such as cowpea, are necessary to enable appropriate management for sustainable cultivation in semiarid conditions. Such studies aim not only to mitigate the effects of drought on these plants, but also to understand the processes involved in recovery and elucidate how stress mitigators, such as silicon and methionine, can contribute to post-rehydration physiological adjustments.

Thus, based on the considerations presented above, this study sought to examine the impact of periods of drought and rehydration on the physiological processes of cowpea. Specifically, we investigated how acclimation responses during water restriction relate to post-stress recovery potential. Furthermore, we evaluated how silicon and methionine could contribute to improvements in biochemical indicators and resource allocation during the drought period, consequently favoring plant recovery. Therefore, the objective of this study was to evaluate the effect of silicon and methionine in optimizing recovery from water stress in cowpea plants of the “BRS Exuberante” variety subjected to periods of water restriction and subsequent rehydration. The hypothesis was that foliar application of silicic acid and/or methionine would be able to modulate plant physiology through changes in biochemical metabolism and growth regulation, thus promoting more efficient recovery after water deficit.

## Materials and methods

### Description of the experiment site

**Fig. 1:**
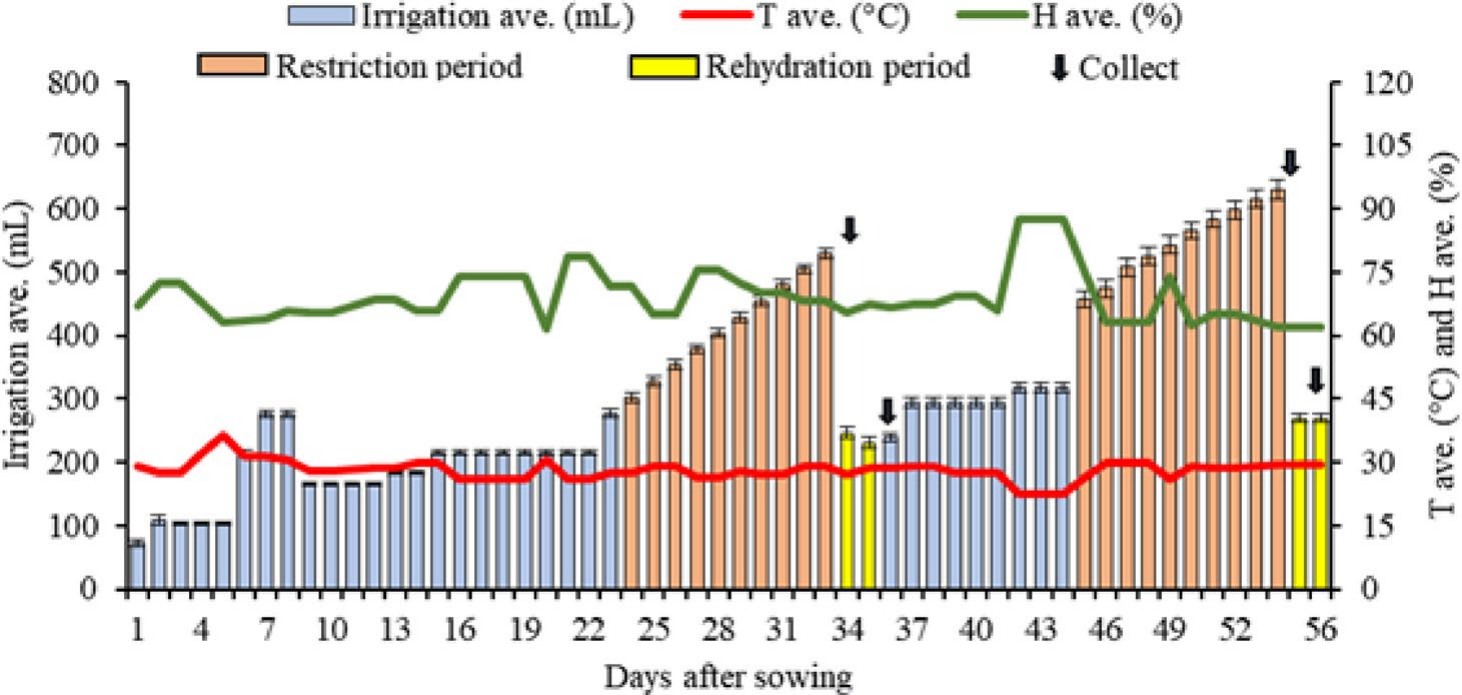
Average temperature in °C (T ave.), average humidity in % (H ave.), and average irrigation in mL (Irrigation ave.) during the experiment. The error bars indicate the standard error (SE, *n* = 16).

This study was conducted in a greenhouse belonging to the Paraíba State University (UEPB) *campus* II, in Lagoa Seca, Paraíba, Brazil (7º09′17″ S, 35º52′16″ W) at an altitude of 652 m. The analyses were performed in the Laboratory of Ecophysiology of Cultivated Plants (EcoLab), located in the Três Marias Complex, of the Center for Biological and Health Sciences of UEPB *campus* I, in Campina Grande, Paraíba, Brazil (07º12′42″ S, 35º54′36″ W), at an altitude of 521 m. The temperature and humidity inside the greenhouse were recorded by an Acurite digital thermo-hygrometer (Humidity Monitor, model 010883), presenting daily temperature averages around 28 °C, with a minimum average of around 20 °C and a maximum average of 36 °C. Humidity reached a daily average of 69%, with the minimum and maximum averages recorded being 40% and 90%, respectively (Fig. 1).

### Experimental design and treatments

The experiment was conducted in a completely randomized design (CRD) in a 2 × 4 factorial arrangement with four replicates. Each replicate corresponded to a pot containing four plants, the number considered necessary for two collections at the V3 and R1 stages. The number of replicates was defined to ensure an adequate estimate of the experimental error within the controlled greenhouse environment. The first factor consisted of two periods: a stress period (irrigation restriction for 10 days) and a rehydration period (irrigation resumption for 2 days). The second factor referred to the elicitor applications, namely: control (no application); silicon (300 mg L^− 1^ Si); methionine (890 mg L^− 1^ Met); and their respective combinations, silicon and methionine (300 mg L^− 1^ Si + 890 mg L^− 1^ Met). The Si concentration was based on previous studies by Silva et al.^16^, Santos et al.^17^ and Araújo et al.^9^, who recommended the use of 300 mg L^− 1^ of Si. For Methionine, a concentration of 890 mg L^− 1^ was used based on the studies by Merwad et al.^18^ and Oliveira et al.^10^. The Si source used was Sifol powder^®^, containing approximately 42% silicon in the form of silicic acid (H_4_SiO_4_), and the methionine used was Dinâmica’s L-Methionine (reagent, PA), containing 98% Met (C_5_H_11_NO_2_S).

### Installation and experimental conduct

To conduct the experiment, 3.6 L polyethylene pots were prepared with a thin layer of gravel at the base and filled with 3.7 kg of dry soil. This soil was previously homogenized to obtain a representative sample of the substrate used. The sample was subsequently sent to the Irrigation and Salinity Laboratory of the Federal University of Campina Grande for analysis of its chemical and physical characteristics, the results of which are described in the supplementary materials. After preparing the pots, they were saturated with water to obtain a substrate close to field capacity. The pots were covered with plastic bags to prevent water evaporation. Twenty-four hours later, the pots were weighed and sown with five seeds per pot, using “BRS Exuberante” cowpea seeds supplied by the Brazilian Agricultural Research Corporation (Embrapa Meio-Norte). These seeds were sorted to eliminate those with physical or biological damage and/or malformations. The pots were arranged on a bench in four rows of four pots, spaced 20 cm apart.

Seven days after sowing, plants were thinned to four per pot. During the experimental period, preventive applications of the insecticide Actara^®^ and the fungicide Amistar^®^ were carried out, following the manufacturer’s recommendations. Topdressing fertilization was also carried out via fertigation with DripSol^®^ MAP-Monoammonium Phosphate (12% nitrogen and 50% phosphorus) and potassium chloride (60% potassium), according to the nutritional needs of the crop, following the proposal of Melo^19^. Twenty-two and forty-three days after sowing, treatments were applied via foliar spraying using a 2-L Dasshaus manual compression sprayer. Applications lasted 20 s, the time required to reach the runoff point on the leaves. Approximately 139 ml was applied per pot, with a standard error of 3 ml (obtained using a flow rate test with 10 pots). For better elicitor adhesion, the Wil Fix^®^ Adhesive spreader was used, following the manufacturer’s instructions. After treatment application, all pots were subjected to irrigation restriction. Ten days after treatment application, the first collection was performed. After this, the pots returned to regular irrigation for two days, where the second and final collection occurred. During the experiment, four collections were performed, two at the V3 stage and the other two at the R1 stage, with each collection consisting of the removal of one plant. In both phenological stages, the first collection was performed after 10 days of water restriction and the other after 2 days of rehydration.

### Irrigation management

Irrigation management was performed daily using the weighing method, as described by Silva et al.^20^, with adaptations. The water evapotranspired the day before each irrigation event was replaced. To determine the reference mass, the substrate was initially saturated with water corresponding to 75% (v/m) of the soil mass and covered with a plastic bag to prevent evaporation. After 24 h, the mass of the pots with the substrate at field capacity was obtained, which was used as a reference for the irrigation events. The pots were weighed daily, and the volume of water corresponding to 70% of the evapotranspiration was replaced. This volume proved to be sufficient for complete cowpea development, as described by Kanda et al.^21^. Weighing and irrigation were performed daily between 7:00 and 8:00 a.m., using a portable digital scale with a capacity of 15 kg (TOMATE, model SF-440). At the end of the experiment, the total volume of water applied was 71 L in the control pots, 70 L in the Si treatment, 74 L in the Met treatment and 67 L in the Si + Met treatment, totaling 282 L throughout the experimental period.

### Variables analyzed

On days 33 and 36 (V3) and 55 and 57 after sowing (R1), the relative water content (RWC) (%) was evaluated as an indicator of water status, following the method proposed by González and González^22^. To verify membrane damage, electrolyte leakage (EL) (%) was evaluated, which indirectly estimates the level of damage to cell membranes, according to Scotti-Campos^23^ and lipid peroxidation (MDA), which was determined by quantifying malonaldehyde, following the method proposed by Cakmak & Host^24^, through the reaction with thiobarbituric acid, and the results expressed in nmol MDA g^− 1^ FM. For the evaluation of soluble proteins and the enzymatic antioxidant mechanism, fully expanded leaves located in the median position of the plant were collected, in which the concentration of total soluble proteins (TSP) was determined by the Bradford method^25^, and the results expressed in mg TSP g^− 1^ FM, and the enzymes superoxide dismutase (SOD), catalase (CAT) and ascorbate peroxidase (APX) followed the same methodology adopted for TSP extraction, however, for the readings of SOD activity was performed by the method proposed by Giannopolitis & Ries^26^, CAT by Kar and Mishra^27^ and APX by Nakano & Asada^28^, and the results expressed in UA g^− 1^ FM, µmol H₂O₂ min^− 1^ mg^− 1^ protein and µM ascorbate min^− 1^ g^− 1^ proteins, respectively.

To verify the osmotic adjustment and osmoprotection indicators, the proline (PRO) content (µmol of PRO g^− 1^ MF) was evaluated using the colorimetric method proposed by Bates et al.^29^. The osmotic adjustment was verified by evaluating the concentration of total soluble sugars (TSS) (mg TSS g^− 1^ FM), determined by the “phenol-sulfuric” method described by Dubois et al.^30^. The quantification of sucrose (SUC) (mg SUC g^− 1^ FM), performed according to the method of Van Handel^31^, using a colorimetric assay with anthrone. The quantification of leaf pigments was performed according to the method of Sims and Gamon^32^, absorbance readings were taken in a spectrophotometer at wavelengths specific for each pigment, after which the absorption values were applied to specific equations for each pigment, namely chlorophyll *a* (Chla), chlorophyll *b* (Chlb), carotenoids (Car), anthocyanins (Ant) and total chlorophylls (total Chl), the results were expressed in mg 100 g^− 1^ FM and later converted to µg 100 g^− 1^ FM.

The growth indicators evaluated were total leaf area (TLA) (cm²) following the method described by Cavalcante et al.^6^ with adaptations, in which the leaflets of the cowpea plants were detached and subsequently digitized using a Poco M4 Pro smartphone, at a scale of two centimeters. The images were labeled correctly, and the measurement was performed using ImageJ software. To measure total dry mass (TDM) (g), the plants had their leaves separated from the branches and were placed separately in properly labeled paper bags. Then, they were placed in an oven with forced air circulation at 65 °C for 72 h to dry. After this period, the plant material was weighed on an analytical balance with an accuracy of 0.0001 g. Since part of the fresh mass (one trifoliate) was removed for biochemical analyses, it was necessary to estimate the dry mass of the removed trifoliate to be added to the final mass. To determine the specific leaf area (SLA), three leaf discs were removed by cutting them with a copper punch. After removal, the discs were stored in paper bags and dried in an oven with controlled air circulation at 65 °C for 48 h to determine the dry mass of the discs. SLA was calculated according to the method described by Silva^33^, and the results were expressed in mm² mg^− 1^.

The leaf area ratio (LAR) (cm² mg^− 1^) and leaf mass ratio (LMR) (g g^− 1^) were determined by the method described by Gorni et al.^34^. Similarly, the leaf area index (LAI) (cm^2^ cm^− 2^) according to Blanco & Folegatti^35^, net assimilatory rate (NAR) (mg cm^− 2^ day^− 1^) according to Díaz-López et al.^36^, crop growth rate (CGR) (mg cm^− 2^ day^− 1^) as proposed by Khan et al.^37^, leaf area duration (LAD) (m² day^− 1^) as proposed by Watson^38^, and water use efficiency (WUE) (g mm^− 1^) were determined according to Meneghetti et al.^39^.

### Statistical analysis

The data obtained were subjected to the Shapiro-Wilk normality test^40^ and Levene’s homogeneity of variance test^41^. Once these premises were met, analysis of variance (ANOVA) was performed using the F test, considering significance at *p* < 0.05. Then, the means between the periods of water restriction and rehydration were compared by Student’s t-test for dependent samples (*p* < 0.05). To complement this analysis, Cohen’s D was also applied for dependent samples^42^ in order to assess the size of the rehydration effect. Cohen’s D is calculated by dividing the mean of the differences by the standard deviation of the differences, resulting in a dimensionless value that indicates the magnitude of the effect, which can be positive or negative. Comparisons between the different elicitors were performed by Tukey’s test (*p* < 0.05). In addition, Pearson correlation analysis was conducted. All analyses were performed in R software (RStudio, version 4.1), using the agricolae, rstatix, effsize, and ggcorrplot packages^43^.

## Results

### Indicator of water status and membrane damage

**Fig. 2:**
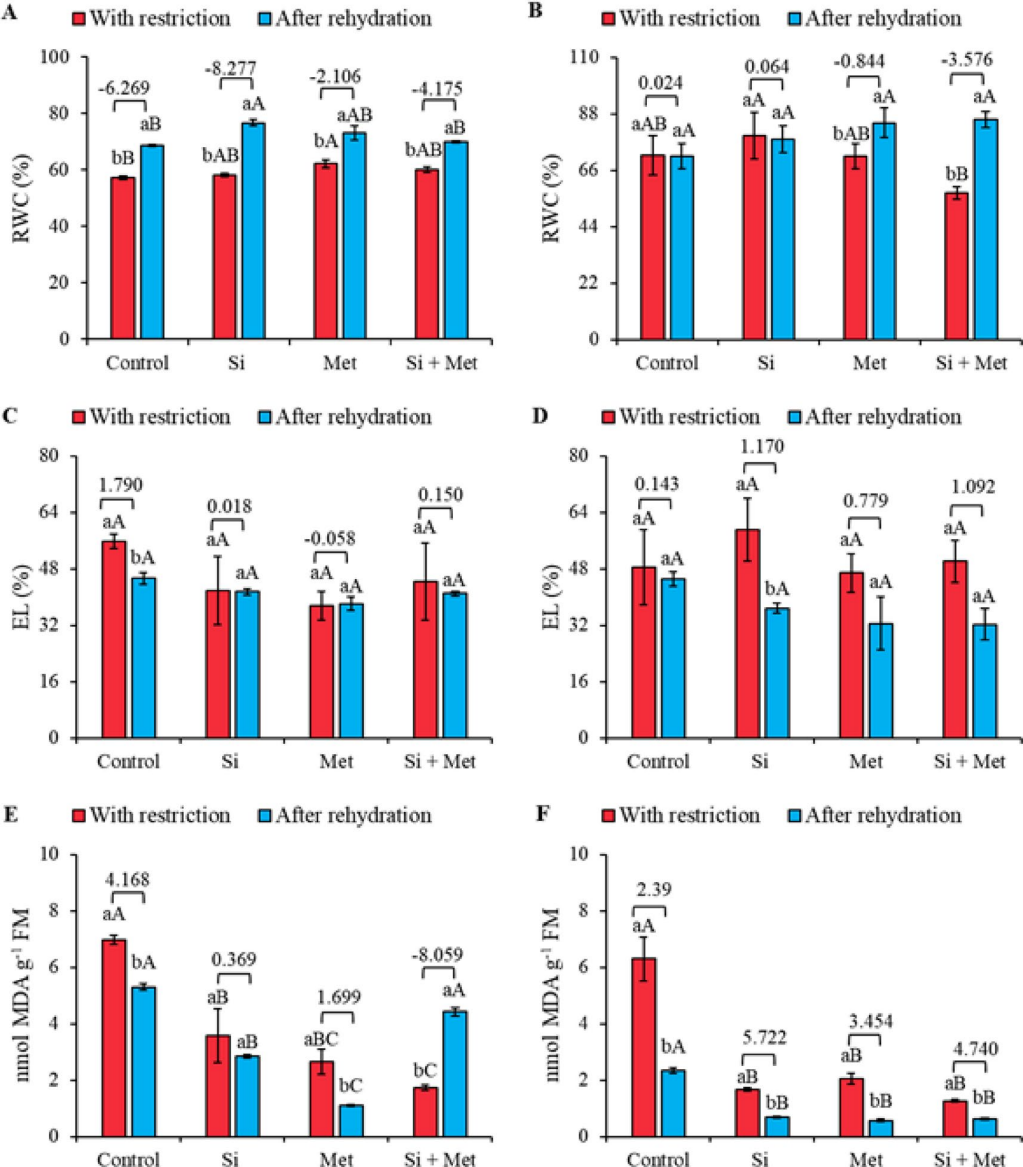
Relative water content (RWC) (**A** and **B**), electrolyte leakage (EL) (**C** and **D**) and lipid peroxidation (MDA) (**E** and **F**) at phenological stages *V3* (A, C and E) and *R1* (B, D and F) in cowpea “BRS Exuberante”, subjected to control treatments (Nothing applied), Si (300 mg L^− 1^ silicon), Met (890 mg L^− 1^ methionine) and Si + Met (300 mg L^− 1^ silicon + 890 mg L^− 1^ methionine) during the water restriction period (10 days) and after rehydration (2 days). Lowercase letters show differences between the restriction and rehydration periods by Student’s test (*p* ≤ 0.05), while uppercase letters show differences between the attenuators by Tukey’s test (*p* ≤ 0.05). The values above the bars represent the effect size by Cohen’s D test, and the error bars indicate the standard error (SE, *n* = 4).

The relative water content (RWC) of cowpea plants cv. “BRS Exuberante” at stage V3 under water restriction decreased significantly (*p* ≤ 0.05). However, treatments with silicon (Si), methionine (Met), and the Si + Met combination provided increases of 1.58%, 8.63%, and 4.96%, respectively, compared to the control (*p* ≤ 0.05) (Fig. 2A). At the same phenological stage, after rehydration, treatments with Si and Met alone promoted increases compared to the control, corresponding to 11.81% and 6.67%, respectively (*p* ≤ 0.05). The Si treatment showed the most significant rehydration effect, evidenced by the effect size of -8.27. At the R1 stage, significant differences between the water restriction and rehydration periods were observed only in the treatments with Met and the Met + Si combination (*p* ≤ 0.05) (Fig. 2B). The Si treatment under water restriction resulted in a 10.88% increase in relative water content compared to the control (*p* ≤ 0.05). After rehydration, no significant differences were observed; however, the Si and Met combination showed a greater rehydration effect, with a value of -3.57.

Electrolyte leakage did not show statistically significant differences between treatments at both phenological stages (*p* ≤ 0.05) (Fig. 2C and D). At stage V3, the highest leakage value was observed in the control during the water restriction period. After rehydration, there was a significant reduction of 18.8% (*p* ≤ 0.05), accompanied by a robust effect size of 1.79 (Fig. 2C). At stage R1, Si treatment promoted more effective recovery, with a significant reduction of 37.7% after rehydration (*p* ≤ 0.05), associated with an effect size of 1.17 (Fig. 2D). Although Met and Si + Met treatments showed no statistical differences after rehydration (*p* ≤ 0.05), both exhibited considerable effect sizes of 0.77 and 1.09, respectively (Fig. 2C and D).

Lipid peroxidation (MDA) of cowpea plants at stage V3 showed significant differences between the periods of water restriction and rehydration in the control and Met treatments, with reductions of 24.1% and 58.7%, respectively (*p* ≤ 0.05) (Fig. 2E). During restriction, treatments with Si, Met, and Si + Met promoted significant reductions compared to the control, corresponding to 48.9%, 62.1%, and 75.3%, respectively (*p* ≤ 0.05). After rehydration, treatments with Si and Met alone maintained significant reductions compared to the control, with values of 46.3 and 79.3%, respectively (*p* ≤ 0.05). The largest rehydration effect sizes were observed in the control (4.16) and Met treatment (1.69) (Fig. 2E). At the R1 stage, MDA levels showed significant reductions during water restriction, being 73.6%, 67.4%, and 79.9% for the Si, Met, and Si + Met treatments, respectively, compared to the control (*p* ≤ 0.05) (Fig. 2F). During rehydration, the reductions compared to the control were 70.8%, 75.9%, and 73.3% for Si, Met, and Si + Met, respectively (*p* ≤ 0.05). When comparing the restriction and rehydration periods, all treatments showed reductions in MDA levels, corresponding to 62.9% in the control, 59.0% in the Si treatment, 72.6% in the Met treatment, and 50.7% in the Si + Met combination (*p* ≤ 0.05). The largest effect sizes were observed in the Si (5.72), Met (3.45), and Si + Met (4.74) treatments (Fig. 2F).

### Soluble proteins and antioxidant enzymes

**Fig. 3:**
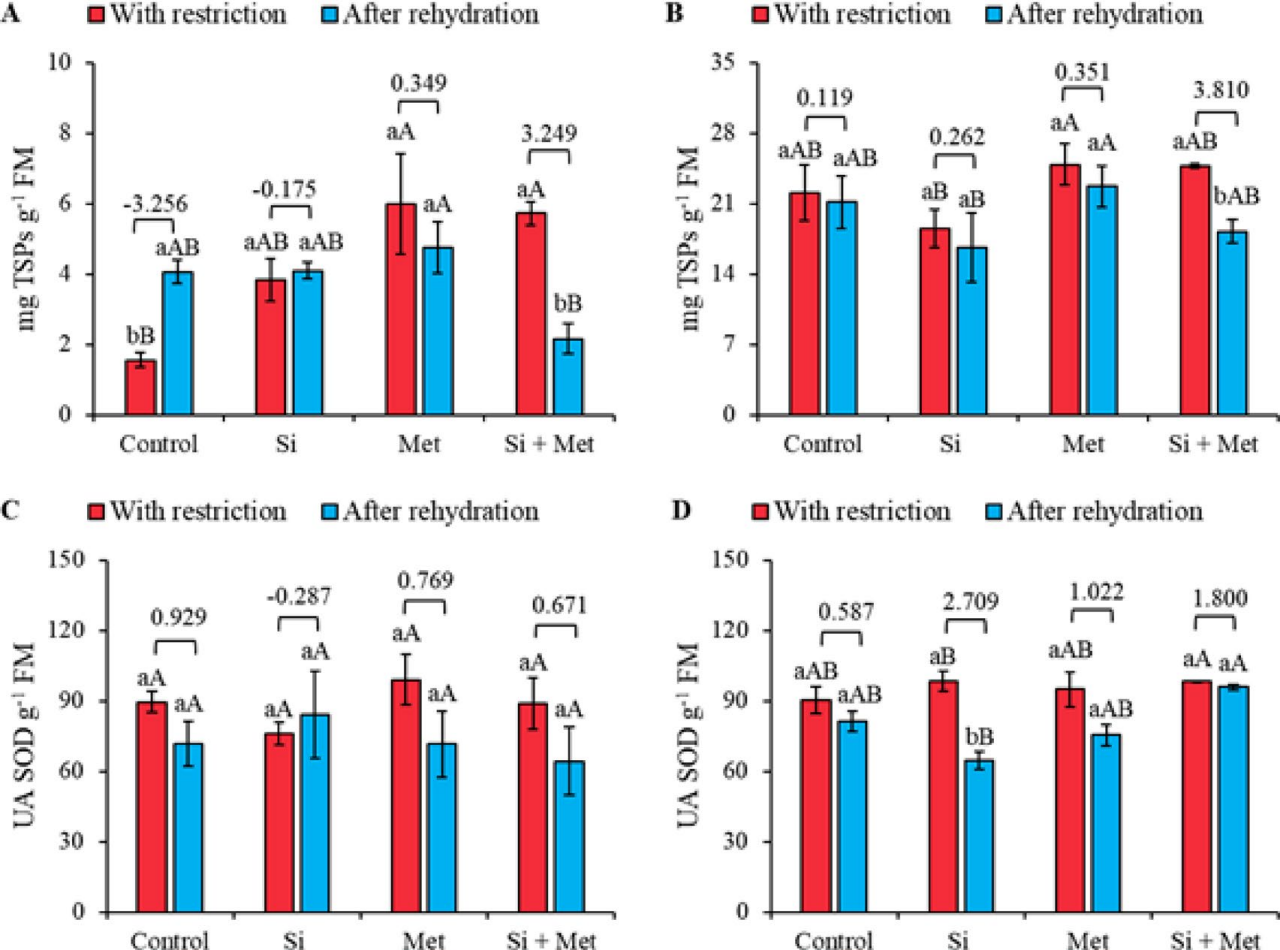
Total soluble proteins (TSP) (**A** and **B**) and superoxide dismutase (SOD) (**C** and **D**) at phenological stages *V3* (A, C and E) and *R1* (B, D and F) in cowpea “BRS Exuberante”, subjected to control treatments (Nothing applied), Si (300 mg L^− 1^ silicon), Met (890 mg L^− 1^ methionine) and Si + Met (300 mg L^− 1^ silicon + 890 mg L^− 1^ methionine) during the water restriction period (10 days) and after rehydration (2 days). Lowercase letters show differences between the restriction and rehydration periods by Student’s test (*p* ≤ 0.05), while uppercase letters show differences between the attenuators by Tukey’s test (*p* ≤ 0.05). The values above the bars represent the effect size by Cohen’s D test, and the error bars indicate the standard error (SE, *n* = 4).

At stage V3, treatments with Si, Met, and the Si + Met combination stimulated increases in total soluble protein (TSP) during water restriction compared to the control, which was also under restriction, corresponding to 147, 287, and 269%, respectively (*p* ≤ 0.05) (Fig. 3A). After rehydration, the Met treatment showed a significant increase of 17.2% compared to the control (*p* ≤ 0.05). Rehydration had a greater impact on the control, evidenced by a significant increase of 161.2% in TSP, accompanied by a substantial effect size of -3.2 (Fig. 3A). During water restriction at stage R1, Met application promoted an increase of 12.2% compared to the control (*p* ≤ 0.05) (Fig. 3B). After rehydration, Met treatment resulted in a 7.5% increase in PST levels compared to the control (*p* ≤ 0.05). The largest effect size was recorded in the treatment with the Si + Met combination (3.8) (Fig. 3B).

Superoxide dismutase (SOD) activity at stage V3 did not show statistically significant differences between the periods of water restriction and rehydration, nor between treatments with different elicitors (Fig. 3C). However, the analysis of effect sizes indicated that rehydration had a greater impact on the control (0.929) and Met (0.769) treatments. Despite the absence of statistical differences, the highest SOD level was recorded in the Met treatment under water restriction (Fig. 3C). At stage R1, SOD activity decreased after rehydration in all treatments. The treatments with Si, Met, and Si + Met stood out, showing the largest effect sizes in this reduction, with values of 2.709 for Si, 1.022 for Met, and 1.800 for Si + Met (Fig. 3D). However, only the Si treatment showed a significant reduction (*p* ≤ 0.05), corresponding to 34.4% after rehydration. During the restriction period, the Si and Si + Met treatments showed the highest SOD levels, with increases of 8.8 and 8.7%, respectively, compared to the control (*p* ≤ 0.05). After rehydration, the Si + Met treatment maintained high SOD levels, with a significant increase of 18.1% compared to the control (*p* ≤ 0.05) (Fig. 3D).

**Fig. 4:**
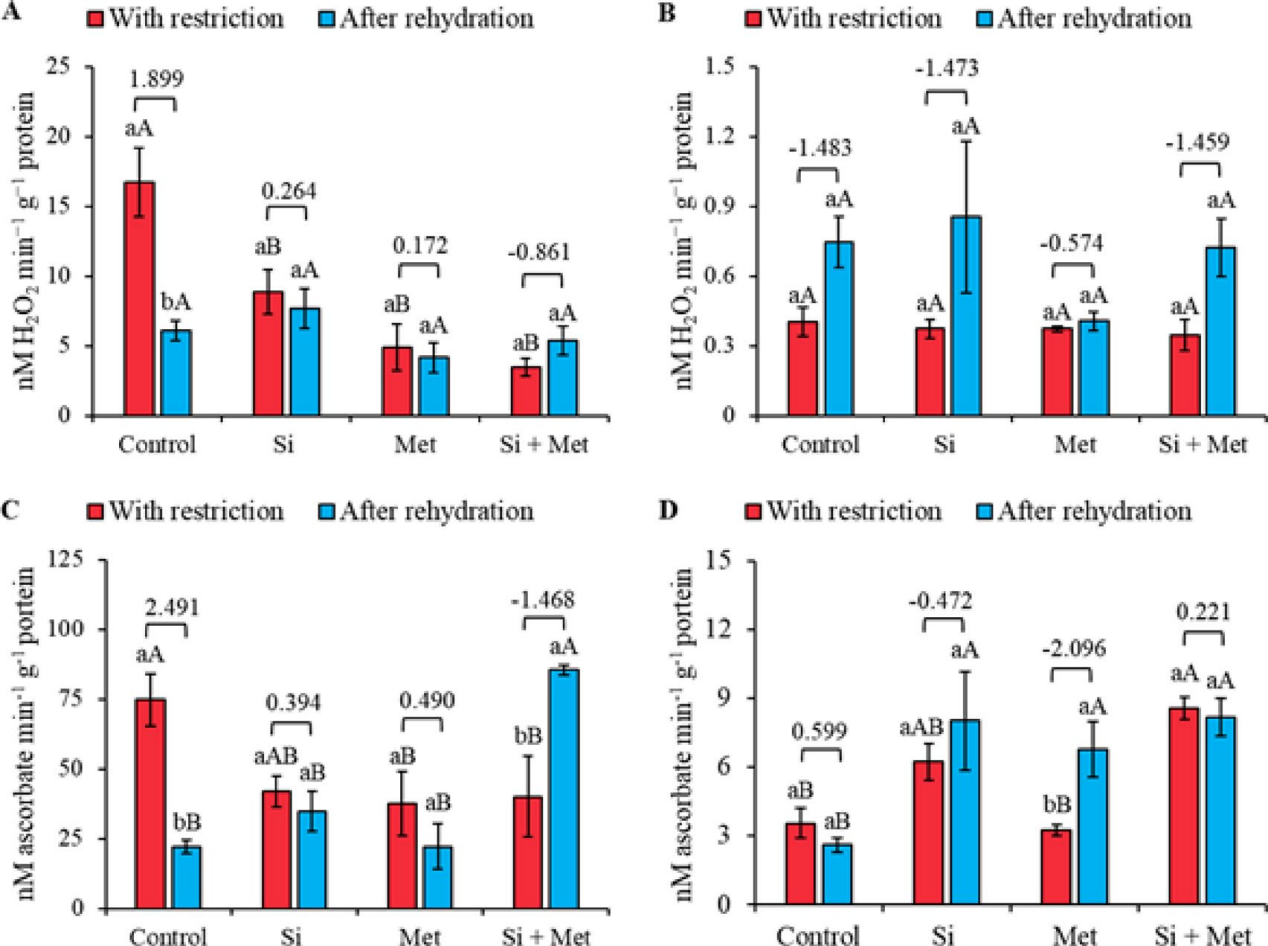
Catalase (CAT) (**A** and **B**) and ascorbate peroxidase (APX) (**C** and **D**) at phenological stages *V3* (A, C and E) and *R1* (B, D and F) in cowpea “BRS Exuberante”, subjected to control treatments (Nothing applied), Si (300 mg L^− 1^ silicon), Met (890 mg L^− 1^ methionine) and Si + Met (300 mg L^− 1^ silicon + 890 mg L^− 1^ methionine) during the water restriction period (10 days) and after rehydration (2 days). Lowercase letters show differences between the restriction and rehydration periods by Student’s test (*p* ≤ 0.05), while uppercase letters show differences between the attenuators by Tukey’s test (*p* ≤ 0.05). The values above the bars represent the effect size by Cohen’s D test, and the error bars indicate the standard error (SE, *n* = 4).

The highest catalase (CAT) activity was observed in control plants during water restriction. After rehydration, there was a significant reduction of 174% (*p* ≤ 0.05), accompanied by the largest effect size recorded (1.899) (Fig. 4A). When comparing the treatments, it was found that CAT activity in the Si, Met, and Si + Met treatments was significantly lower during restriction, with reductions of 47.3, 71.2, and 79.6%, respectively, compared to the control under the same conditions (*p* ≤ 0.05) (Fig. 4A). At the R1 stage, no statistical differences were observed between the periods of water restriction and rehydration, nor between the treatments (Fig. 4B). However, a general trend of increased CAT levels was observed after rehydration in all treatments, with the highest activity recorded in the Si treatment. The largest effect sizes were observed in the control (1.483), Si (-1.473) and Si + Met (-1.459) (Fig. 4B).

The highest ascorbate peroxidase (APX) activities at the V3 stage were observed in the control under water restriction and in the Si + Met treatment after rehydration, both significantly different from the other treatments (*p* ≤ 0.05). Rehydration in the Si + Met treatment resulted in a significant increase of 113.4% (*p* ≤ 0.05), accompanied by the largest effect size recorded (-1.468) (Fig. 4C). At the reproductive stage (R1), APX levels increased significantly after rehydration compared to the control in the same period, corresponding to 220% for the Si treatment, 168% for Met and 226.4% for Si + Met (*p* ≤ 0.05). During water restriction, the Si and Si + Met treatments also showed significant increases in relation to the control, with 76.2 and 141.1%, respectively (*p* ≤ 0.05). Only in the Met treatment was a significant difference observed between the restriction and rehydration periods (*p* ≤ 0.05), accompanied by the largest effect size recorded (-2.096) (Fig. 4D).

### Indicators of osmoprotection and osmoregulation

**Fig. 5:**
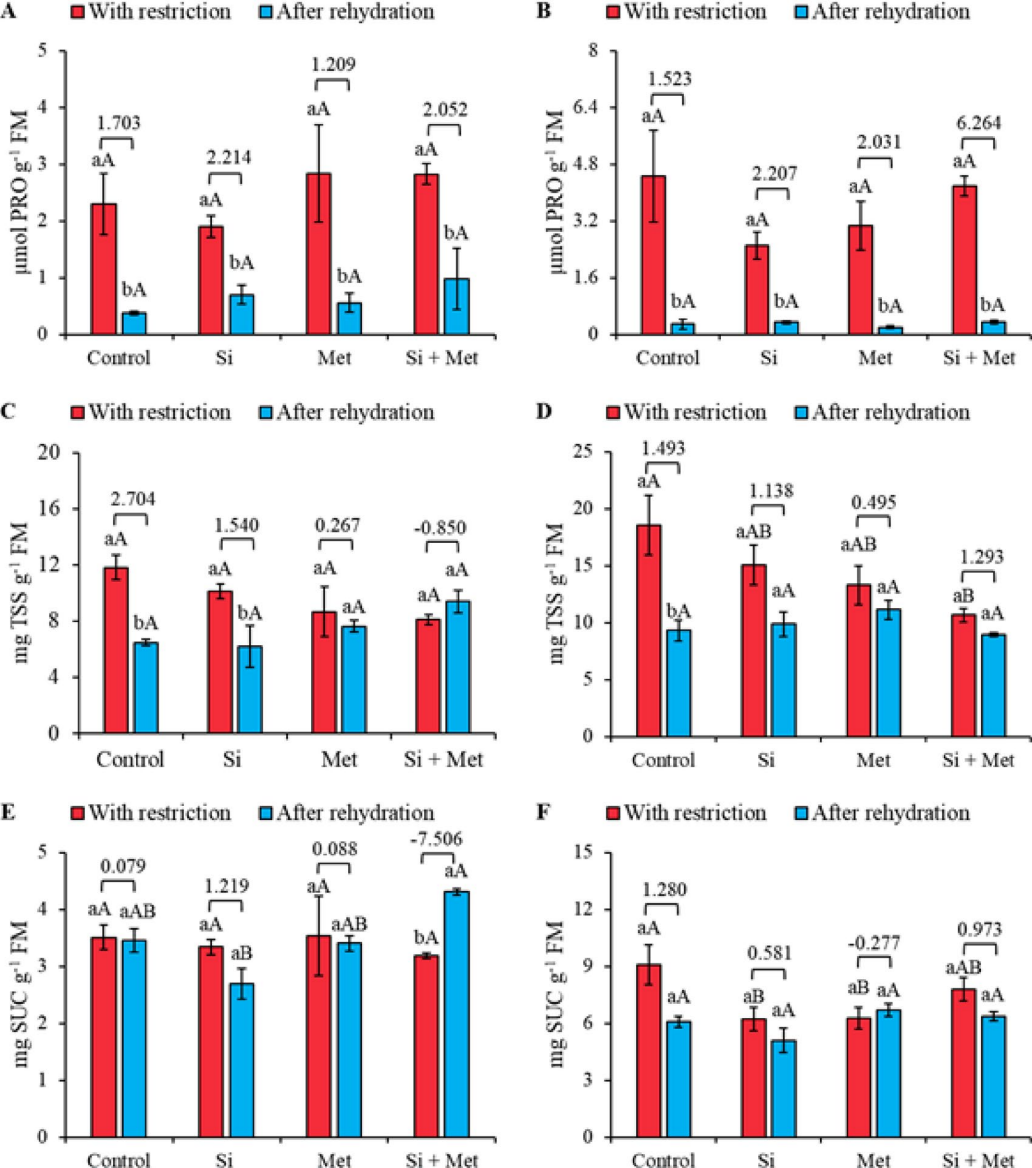
Proline (PRO) (**A** and **B**), total soluble sugars (TSS) (**C** and **D**) and sucrose (SUC) (**E** and **F**) at phenological stages *V3* (A, C and E) and *R1* (B, D and F) in cowpea “BRS Exuberante”, subjected to control treatments (Nothing applied), Si (300 mg L^− 1^ silicon), Met (890 mg L^− 1^ methionine) and Si + Met (300 mg L^− 1^ silicon + 890 mg L^− 1^ methionine) during the water restriction period (10 days) and after rehydration (2 days). Lowercase letters show differences between the restriction and rehydration periods by Student’s test (*p* ≤ 0.05), while uppercase letters show differences between the attenuators by Tukey’s test (*p* ≤ 0.05). The values above the bars represent the effect size by Cohen’s D test, and the error bars indicate the standard error (SE, *n* = 4).

Proline (PRO) levels at the V3 stage increased significantly under water restriction (*p* ≤ 0.05) (Fig. 5A). After rehydration, a significant reduction was observed in all treatments, corresponding to 86.3% in the control (effect size 1.703), 68.4% in the Si treatment (2.214), 82.1% in the Met treatment (1.209), and 67.8% in the Si + Met combination (2.052) (*p* ≤ 0.05) (Fig. 5A). Despite these reductions, no significant differences were detected between treatments, either under water restriction or after rehydration. At the reproductive stage (R1), the behavior was similar to that observed at the vegetative stage (Fig. 5B). There were no significant differences between treatments, regardless of the period. After rehydration, all treatments showed marked reductions in PRO levels, corresponding to 95.4% in the control, 88.0% in the Si treatment, 93.3% in the Met treatment, and 92.6% in the Si + Met combination (*p* ≤ 0.05). These declines were accompanied by large effect sizes: 1.523, 2.207, 2.031, and 6.264 for control, Si, Met, and Si + Met, respectively (Fig. 5B).

At the V3 stage, total soluble sugar (TSS) levels did not show statistically significant differences between treatments, either under water restriction or after rehydration (Fig. 5C). However, when comparing the periods, it was found that the control and Si treatments showed significant reductions in TSS levels, corresponding to 45.7 and 39.6%, respectively (*p* ≤ 0.05), accompanied by effect sizes of 2.704 and 1.540 (Fig. 5C). At the reproductive stage, plants in the control under water restriction showed greater TSS accumulation, significantly higher than the other treatments under the same conditions (Fig. 5D). Among the treatments, the Si + Met combination promoted a significant reduction in TSS levels compared to the control, with a decline of 42.7% (*p* ≤ 0.05). After rehydration, the control showed a significant 49.7% reduction in TSS levels (*p* ≤ 0.05), while all treatments became statistically similar. Analysis of effect sizes generally indicated that soluble sugar levels decreased after rehydration (Fig. 5D).

At the V3 stage, sucrose (SUC) levels were not significantly influenced by the treatments under water restriction (Fig. 5E). However, after rehydration, the combined Si + Met treatment promoted a significant increase of 26.4% (*p* ≤ 0.05) compared to the control, also under rehydration. Furthermore, Si + Met was the only treatment to show an increase in sucrose levels after rehydration, with an increase of 38.7% (*p* ≤ 0.05), accompanied by a significant effect size of -7.506 (Fig. 5E). At the reproductive stage (R1), the control showed greater sucrose accumulation under water restriction, statistically higher than the other treatments (*p* ≤ 0.05) (Fig. 5F). After rehydration, sucrose levels became statistically similar among all treatments. Although no significant differences were observed between the restriction and rehydration periods, the effect sizes indicate that, as in the vegetative stage, sucrose levels tend to decrease after rehydration (Fig. 5F).

### Leaf pigments

**Fig. 6:**
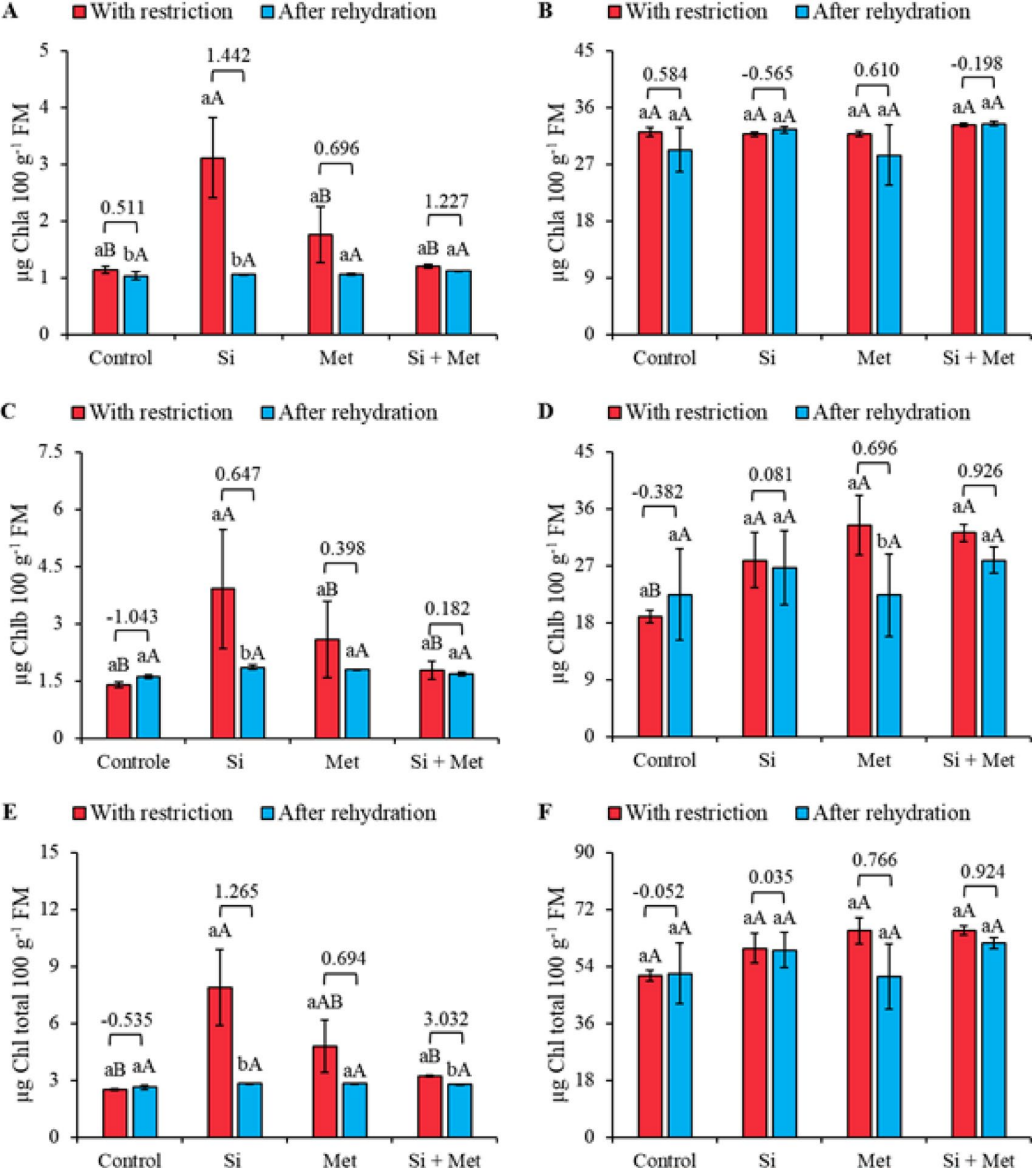
Chlorophyll *a* (Chl a) (**A** and **B**), Chlorophyll *b* (Chl b) (**C** and **D**) and total chlorophyll (Total Chl) (**E** and **F**) at phenological stages *V3* (A, C and E) and *R1* (B, D and F) in cowpea “BRS Exuberante”, subjected to control treatments (Nothing applied), Si (300 mg L^− 1^ silicon), Met (890 mg L^− 1^ methionine) and Si + Met (300 mg L^− 1^ silicon + 890 mg L^− 1^ methionine) during the water restriction period (10 days) and after rehydration (2 days). Lowercase letters show differences between the restriction and rehydration periods by Student’s test (*p* ≤ 0.05), while uppercase letters show differences between the attenuators by Tukey’s test (*p* ≤ 0.05). The values above the bars represent the effect size by Cohen’s D test, and the error bars indicate the standard error (SE, *n* = 4).

At stage V3, only the Si treatment significantly increased chlorophyll a levels under water restriction, representing a 182% increase compared to the control, also under water restriction (*p* ≤ 0.05) (Fig. 6A). After rehydration, no statistically significant differences were observed between treatments; however, the Si treatment showed a 67.7% reduction in chlorophyll *a* levels after rehydration (*p* ≤ 0.05) (Fig. 6A). At stage R1, chlorophyll *a* levels remained statistically similar between treatments and between the restriction and rehydration periods (Fig. 6B).

At the V3 stage, the Si treatment promoted a significant 178% increase in chlorophyll *b* levels compared to the control, both under water restriction (*p* ≤ 0.05). After rehydration, all treatments showed statistically similar levels. Only the Si treatment showed a significant difference between the restriction and rehydration periods, with a 53.8% reduction in chlorophyll *b* levels (*p* ≤ 0.05) (Fig. 6C). At the reproductive stage, the water restriction treatments caused significant increases in chlorophyll b levels compared to the control, corresponding to 47.0, 76.1, and 69.8% for Si, Met, and Si + Met, respectively (*p* ≤ 0.05). After rehydration, chlorophyll *b* levels became statistically similar between treatments. Only the Met treatment showed a significant reduction between the restriction and rehydration periods, with a decrease of 33% (*p* ≤ 0.05) (Fig. 6D).

At stage V3, treatments with Si and Met, applied alone, promoted significant increases in total chlorophyll, corresponding to 212 and 88% compared to the control, both under water restriction (*p* ≤ 0.05) (Fig. 6E). After rehydration, no statistically significant differences were observed between treatments. Between the restriction and rehydration periods, only the Si and Si + Met treatments showed substantial differences, with reductions of 64.1 and 15.6%, respectively (*p* ≤ 0.05) (Fig. 6E). At stage R1, total chlorophyll levels were statistically similar between treatments and between the restriction and rehydration periods (Fig. 6F). However, as observed at stage V3, total chlorophyll levels tended to decrease after rehydration, as indicated by the effect sizes (Fig. 6E and F).

**Fig. 7:**
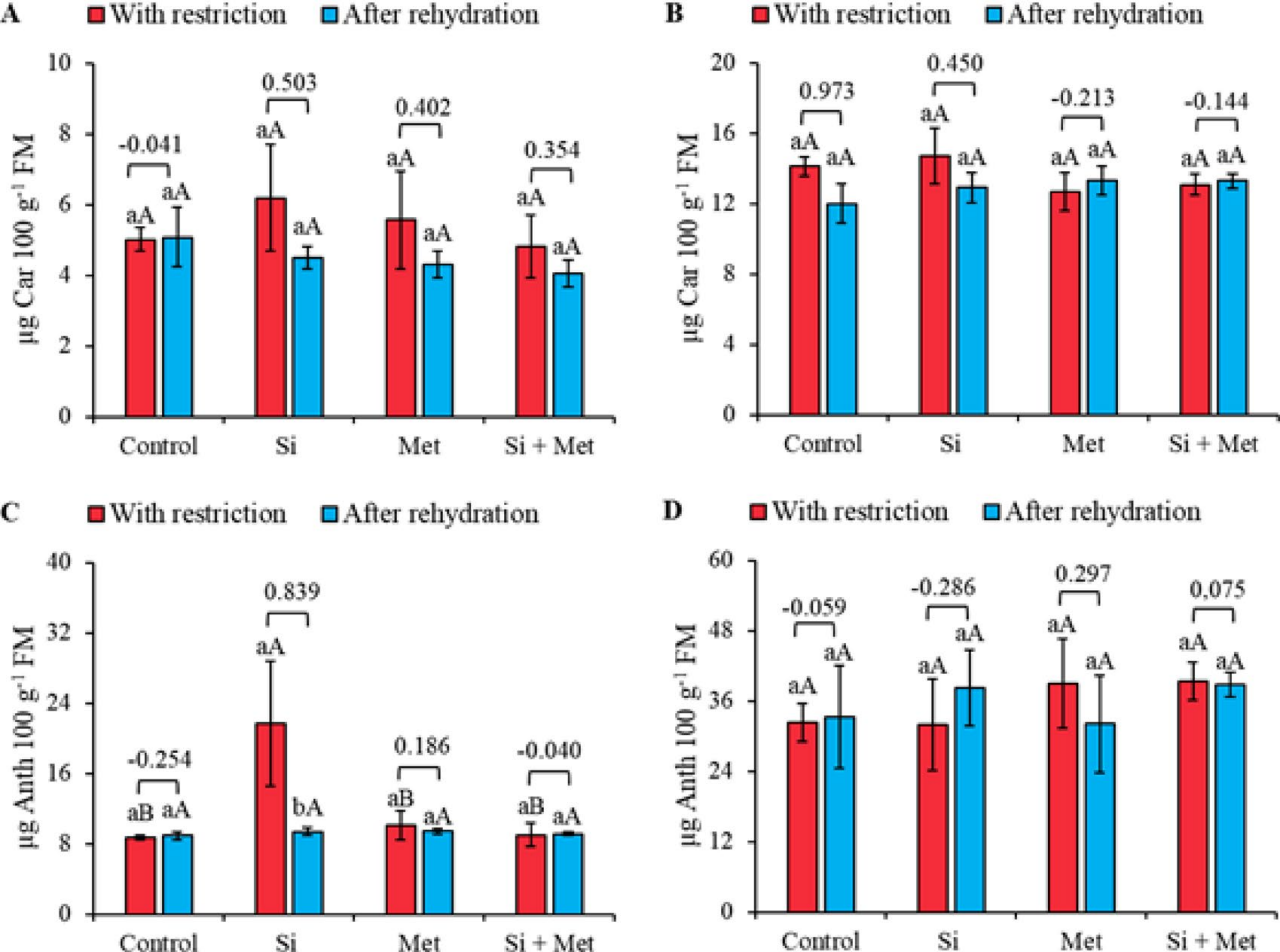
Carotenoids (Car) (**A** and **B**) and Anthocyanins (Anth) (**C** and **D**) at phenological stages *V3* (A, C and E) and *R1* (B, D and F) in cowpea “BRS Exuberante”, subjected to control treatments (Nothing applied), Si (300 mg L^− 1^ silicon), Met (890 mg L^− 1^ methionine) and Si + Met (300 mg L^− 1^ silicon + 890 mg L^− 1^ methionine) during the water restriction period (10 days) and after rehydration (2 days). Lowercase letters show differences between the restriction and rehydration periods by Student’s test (*p* ≤ 0.05), while uppercase letters show differences between the attenuators by Tukey’s test (*p* ≤ 0.05). The values above the bars represent the effect size by Cohen’s D test, and the error bars indicate the standard error (SE, *n* = 4).

Carotenoid (Car) levels were not significantly influenced by elicitors or by the restriction and rehydration periods, either at the V3 (Fig. 7A) or R1 (Fig. 7B) stages. However, the highest Car values ​​were recorded during water restriction, especially in the silicon treatment at both phenological stages (Fig. 7A and B). Furthermore, the effect size analysis indicated a general trend of reduced carotenoid levels after rehydration. At the V3 stage, anthocyanin levels were significantly influenced by the silicon treatment, which promoted a 152.3% increase compared to the control under water restriction (*p* ≤ 0.05) (Fig. 7C). After rehydration, this same treatment showed a significant reduction in anthocyanin levels, corresponding to 57.1% (*p* ≤ 0.05), accompanied by the largest effect size recorded for the variable. At stage R1, however, no significant differences were observed between the restriction and rehydration periods or between the treatments evaluated (Fig. 7D).

### Indicators of adjustment in growth and biomass allocation

**Fig. 8:**
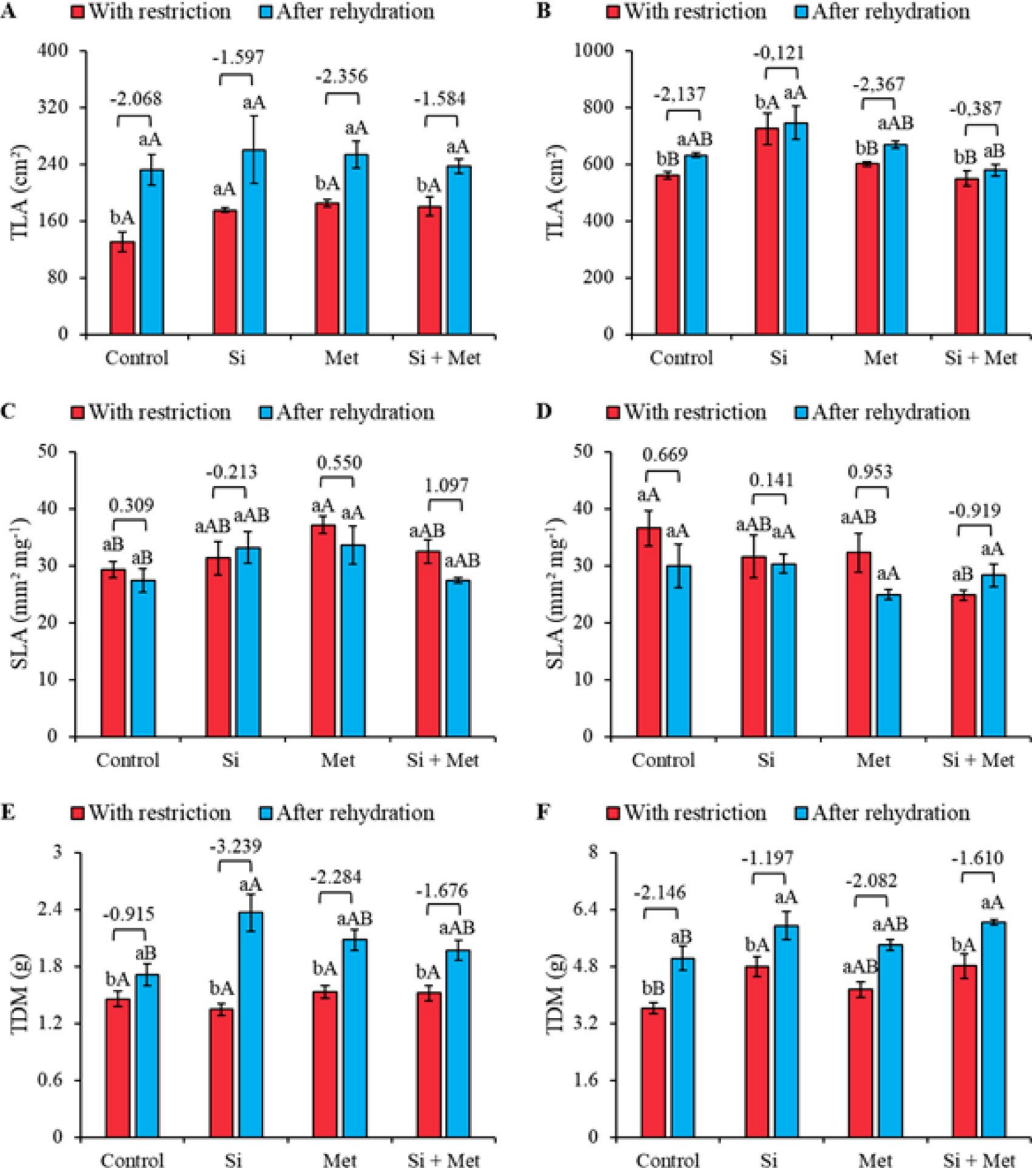
Total leaf area (TLA) (**A** and **B**), Specific leaf area (SLA) (**C** and **D**) and Total dry mass (TDM) (**E** and **F**) at phenological stages *V3* (A, C and E) and *R1* (B, D and F) in cowpea “BRS Exuberante”, subjected to control treatments (Nothing applied), Si (300 mg L^− 1^ silicon), Met (890 mg L^− 1^ methionine) and Si + Met (300 mg L^− 1^ silicon + 890 mg L^− 1^ methionine) during the water restriction period (10 days) and after rehydration (2 days). Lowercase letters show differences between the restriction and rehydration periods by Student’s test (*p* ≤ 0.05), while uppercase letters show differences between the attenuators by Tukey’s test (*p* ≤ 0.05). The values above the bars represent the effect size by Cohen’s D test, and the error bars indicate the standard error (SE, *n* = 4).

In the vegetative stage, total leaf area (TLA) increased after rehydration, as evidenced by the effect sizes, with emphasis on the Met treatment, which recorded the highest value (-2.356) (Fig. 8A). In both periods, restriction and rehydration, no significant differences were observed between treatments. However, when comparing the periods, the control, Met, and Si + Met treatments showed significant increases of 79.2, 37.1, and 31.5%, respectively (*p* ≤ 0.05) (Fig. 8A). At the R1 stage, the silicon treatment stood out for promoting greater TLA both under restriction and after rehydration, with increases of 29.1 and 18.1%, respectively, compared to the control in each period (*p* ≤ 0.05) (Fig. 8B). All treatments showed significant increases in TLA between periods, ranging from 2.8% (Si) to 12.3% (control), while Met and Si + Met recorded increases of 11.3 and 5.2%, respectively (*p* ≤ 0.05). Furthermore, the R1 stage after rehydration was also marked by an increase in TLA indicated by the effect sizes, with emphasis again on the Met treatment, which presented the highest value (-2.356) (Fig. 8B).

In the vegetative stage, the specific leaf area (SLA) showed a significant increase with the application of Si, Met, and Si + Met compared to the control, both under water restriction and after rehydration (Fig. 8C). Under restriction, the increases recorded were 6.8% (Si), 26.6% (Met), and 10.5% (Si + Met) (*p* ≤ 0.05). After rehydration, increases of 21.2% (Si), 23.0% (Met), and 0.2% (Si + Met) were observed compared to the control within each period (*p* ≤ 0.05). No statistical differences were observed between the restriction and rehydration periods (Fig. 8C). At the R1 stage, no statistical differences were observed between the restriction and rehydration periods (Fig. 8D). However, under restriction, the control presented the highest SLA, statistically differentiating itself from the other treatments (*p* ≤ 0.05). After rehydration, however, there were no significant differences between treatments (Fig. 8D).

**Fig. 9:**
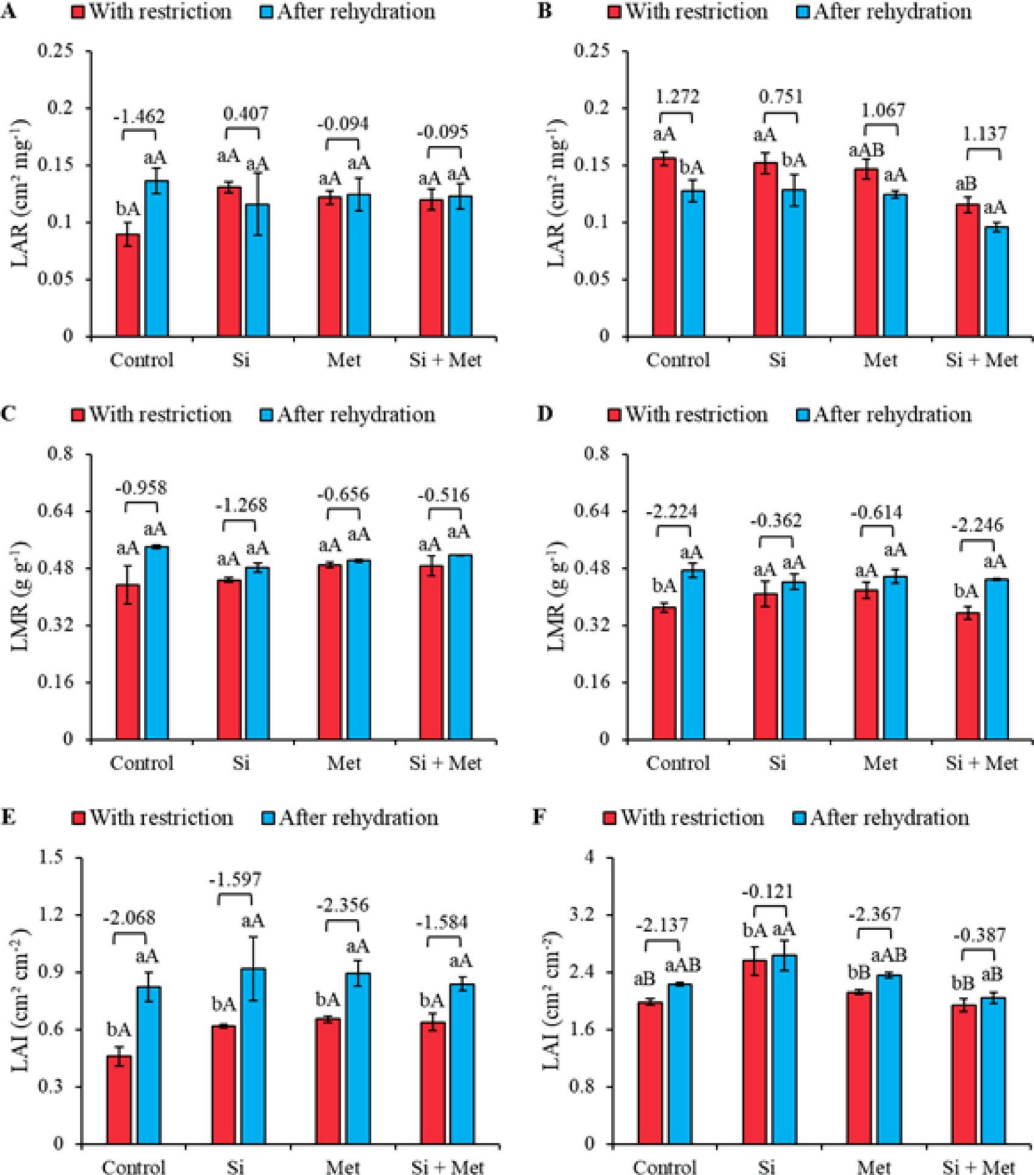
Leaf area ratio (LAR) (**A** and **B**), Leaf mass ratio (LMR) (**C** and **D**) and Leaf area index (LAI) (**E** and **F**) at phenological stages *V3* (A, C and E) and *R1* (B, D and F) in cowpea “BRS Exuberante”, subjected to control treatments (Nothing applied), Si (300 mg L^− 1^ silicon), Met (890 mg L^− 1^ methionine) and Si + Met (300 mg L^− 1^ silicon + 890 mg L^− 1^ methionine) during the water restriction period (10 days) and after rehydration (2 days). Lowercase letters show differences between the restriction and rehydration periods by Student’s test (*p* ≤ 0.05), while uppercase letters show differences between the attenuators by Tukey’s test (*p* ≤ 0.05). The values above the bars represent the effect size by Cohen’s D test, and the error bars indicate the standard error (SE, *n* = 4).

At stage V3, total biomass (TDM) did not show significant differences among elicitors under water restriction. However, after rehydration, treatments with Si, Met, and Si + Met promoted increases of 35.2, 17.6, and 11.7%, respectively, compared to the rehydrated control (*p* ≤ 0.05) (Fig. 8E). When comparing the periods, it was found that all treatments significantly increased TDM, with increases of 21.4% (control), 76.9% (Si), 33.3% (Met), and 26.6% (Si + Met) (*p* ≤ 0.05). The effect sizes confirm this trend, evidencing that Si, Met, and Si + Met had the greatest influence on TDM recovery after rehydration (Fig. 8E). At the R1 stage, the Si, Met, and Si + Met treatments showed higher TDM compared to the control, even under water restriction, with increases of 30.5% (Si), 13.8% (Met), and 33.3% (Si + Met) (*p* ≤ 0.05) (Fig. 8F). After rehydration, these treatments maintained their superiority over the control, with increases of 18.0% (Si), 6.0% (Met), and 20.0% (Si + Met) (*p* ≤ 0.05). Furthermore, between the periods, the control and the Si and Si + Met treatments showed significant increases in TDM, of 38.8, 25.5, and 25.0%, respectively (*p* ≤ 0.05). The leaf area ratio (LAR) at the V3 stage did not show significant differences between treatments or between the restriction and rehydration periods (Fig. 9A). However, the effect size values indicate a trend of increased LAR after rehydration. At the reproductive stage, no statistical differences were observed between treatments after rehydration (Fig. 9B). Only the control and Si treatments showed significant differences between periods, with reductions of 18.0% and 15.8%, respectively (*p* ≤ 0.05) (Fig. 9B).

The leaf mass ratio (LMF) at the vegetative stage did not show significant differences between treatments or between the restriction and rehydration periods. However, the effect size values ​​indicated a trend toward an increase in LMF after rehydration (Fig. 9C). At the R1 stage, no significant differences were observed between treatments either under restriction or after rehydration (Fig. 9D). However, when comparing the periods, it was found that the control and Si + Met treatments showed significant increases in LMF after rehydration, corresponding to 27.0% and 25.7%, respectively (*p* ≤ 0.05). These results were reinforced by the high effect sizes, -2.224 and − 2.246, respectively (Fig. 9D).

At the V3 stage, no statistically significant differences in the leaf area index (LAI) were observed between treatments, either under water restriction or after rehydration (Fig. 9E). However, after rehydration, all treatments showed significant increases in LAI, corresponding to 82.2% (control), 49.1% (Si), 36.9% (Met), and 31.7% (Si + Met) (*p* ≤ 0.05). The effect sizes reinforce this interpretation, indicating a clear trend of increasing LAI after rehydration (Fig. 9E). In the reproductive phase, the treatment with Si alone promoted significant increases compared to the other treatments under water restriction, equivalent to 31.5% in relation to the control (*p* ≤ 0.05) (Fig. 9F). After rehydration, Si maintained superior performance, with an increase of 18.1% compared to the control (*p* ≤ 0.05). Furthermore, the Si, Met, and Si + Met treatments promoted significant increases in LAI between periods, with increases of 4.0, 9.5, and 7.3%, respectively (*p* ≤ 0.05). As in the vegetative stage, the effect sizes indicated a trend of increase in LAI after rehydration (Fig. 9E and F).

**Fig. 10:**
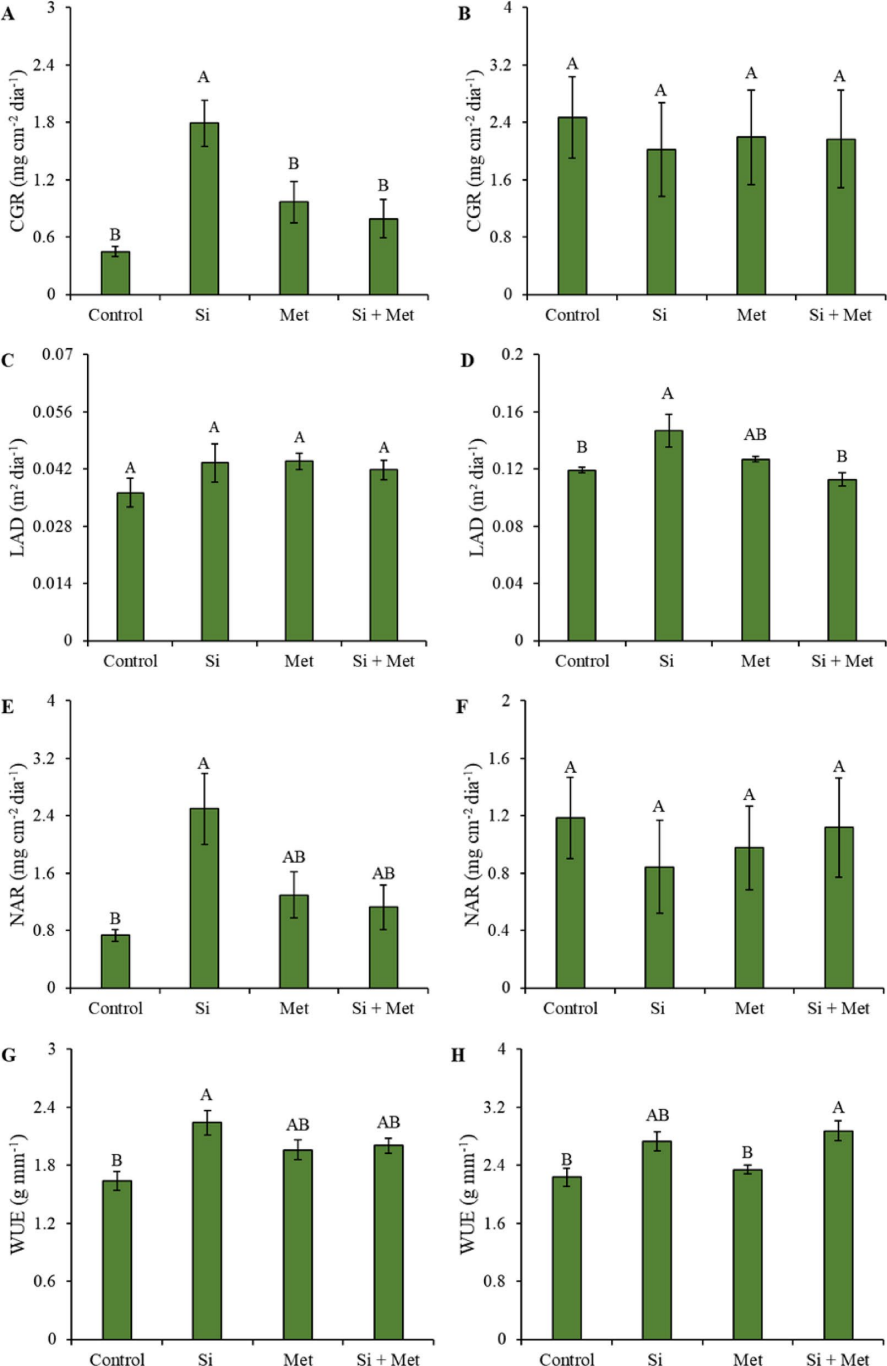
Crop Growth Rate (CGR) (**A** and **B**), Leaf Area Duration (LAD) (**C** and **D**), Net Assimilatory Rate (NAR) (**E** and **F**) and Water Use Efficiency (WUE) (**G** and **H**) at phenological stages *V3* (A, C, E and G) and *R1* (B, D, F and H) in cowpea “BRS Exuberante”, subjected to control treatments (Nothing applied), Si (300 mg L^− 1^ silicon), Met (890 mg L^− 1^ methionine) and Si + Met (300 mg L^− 1^ silicon + 890 mg L^− 1^ methionine) during the water restriction period (10 days) and after rehydration (2 days). Lowercase letters show differences between the restriction and rehydration periods by Student’s test (*p* ≤ 0.05), while uppercase letters show differences between the attenuators by Tukey’s test (*p* ≤ 0.05). The values above the bars represent the effect size by Cohen’s D test, and the error bars indicate the standard error (SE, *n* = 4).

Crop growth rate (CGR) showed a significant increase in the treatment with silicon alone, corresponding to a 325% increase compared to the control (*p* ≤ 0.05) (Fig. 10A). However, at the reproductive stage, no statistical differences in CGR were observed between treatments (Fig. 10B). Similarly, leaf area duration (LAD) did not show significant differences at the V3 stage (Fig. 10C). At the reproductive stage, however, treatments with isolated application of silicon and methionine promoted significant increases compared to the control, with increases of 27.2 and 14.5%, respectively (*p* ≤ 0.05) (Fig. 10D). When evaluating the net assimilation rate (NAR) in the vegetative stage, it was found that the treatments with Si, Met, and Si + Met promoted significant increases in relation to the control, corresponding to 242.8, 71.4, and 57.1%, respectively (*p* ≤ 0.05) (Fig. 10E). At the R1 stage, however, no significant differences were observed between the elicitors (Fig. 10E and F). Similarly, the water use efficiency (WUE) at the V3 stage showed significant increases in the treatments with Si, Met, and Si + Met, equivalent to 37.5, 18.7, and 25%, respectively, in comparison to the control (*p* ≤ 0.05) (Fig. 10G). At the R1 stage, treatments with Si and Si + Met also promoted significant increases in WUE, of 22.7 and 27.2%, respectively, in relation to the control (*p* ≤ 0.05) (Fig. 10H).

### Pearson correlation matrix for silicon and methionine treatments after rehydration in the stadium V3

**Fig. 11:**
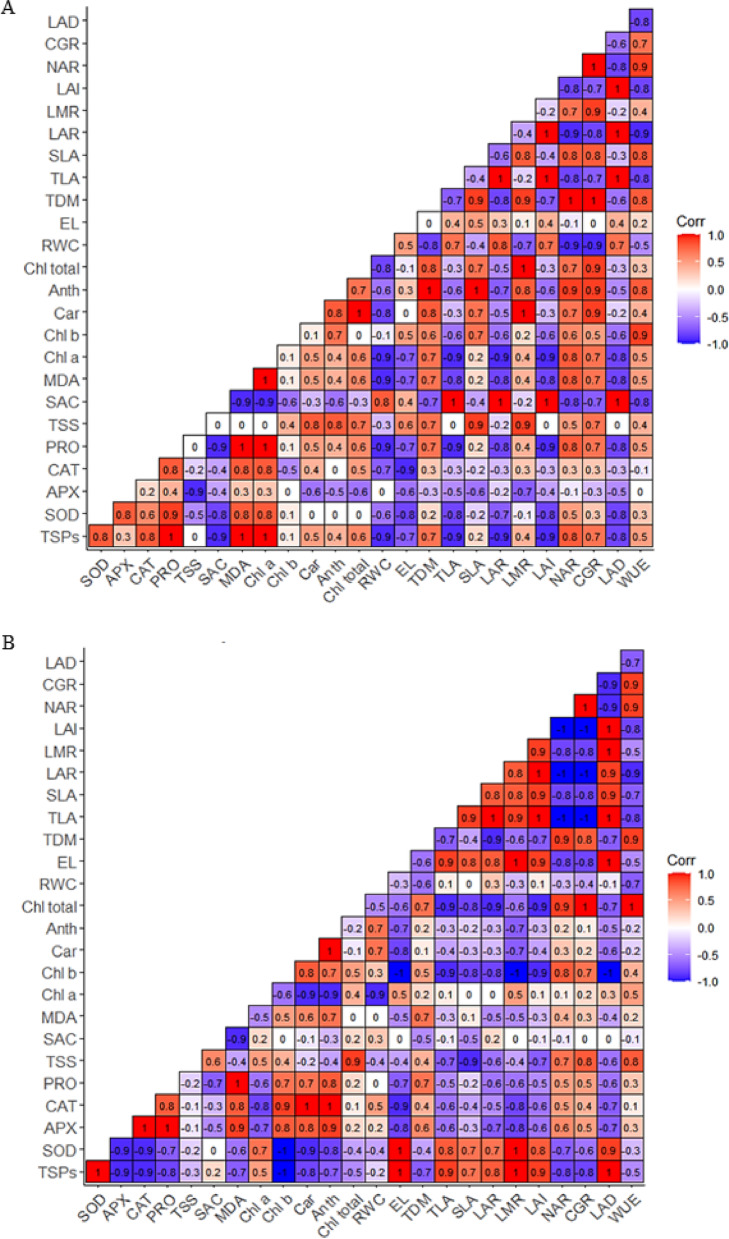
Correlation matrix of Total soluble protein (TSP), Superoxide dismutase (SOD), Catalase (CAT), Ascorbate peroxidase (APX), Proline (PRO), Total soluble sugars (TSS), Sucrose (SUC), Lipid peroxidation (MDA), Chlorophyll *a* (Chl a), Chlorophyll *b* (Chl b), Carotenoids (Car), Anthocyanins (Anth), Total chlorophyll (Chl total), Relative water content (RWC), Electrolyte leakage (EL), Total dry mass (TDM), Total leaf area (TLA), Specific leaf area (SLA), Leaf area ratio (LAR), Leaf mass ratio (LMR), Leaf area index (LAI), Net assimilatory rate (NAR), Crop growth rate (CGR), Leaf area duration (LAD) and Water use efficiency (WUE) of cowpea plants “BRS Exuberante” at the *V3* stage, treated with silicon (**A**) and methionine (**B**) after rehydration.

The Pearson correlation between cowpea plants treated with silicon alone, after rehydration, at the V3 phenological stage, is shown in Fig. 11A. The enzyme superoxide dismutase (SOD) showed a positive correlation with other enzymes of the antioxidant system, such as ascorbate peroxidase (APX) and catalase (CAT). Similarly, SOD, APX, and CAT also showed a positive correlation with total soluble protein (TSP) content. Regarding TSP, a positive correlation was observed with the net assimilatory rate (NAR) and crop growth rate (CGR). The photosynthetic pigments chlorophyll *a* (Chla), chlorophyll *b* (Chlb), carotenoids (Car), and anthocyanins (Anth) showed a positive correlation with total dry matter (TSM). NAR and water use efficiency (WUE) also showed a positive correlation with all evaluated pigments (Chla, Chlb, total Chl, Car, and Anth). On the other hand, lipid peroxidation (MDA) showed a negative correlation with relative water content (RWC), total leaf area (AFT), leaf area ratio (LAR), leaf area index (LAI), and leaf area duration (LAD).

Regarding Pearson’s correlation under isolated methionine application, it was observed that the enzyme superoxide dismutase (SOD) showed a negative correlation with the other antioxidant enzymes, catalase (CAT) and ascorbate peroxidase (APX), and a positive correlation with total soluble protein content (TSP). TSP, in turn, demonstrated a strong positive correlation with variables associated with growth and biomass allocation, such as total leaf area (TLA), specific leaf area (SLA), leaf area ratio (LAR), leaf mass ratio (LMR), leaf area index (LAI), and leaf area duration (LAD). Regarding leaf pigments, it was observed that both carotenoids (Car) and anthocyanins (Anth) showed a positive correlation with relative water content (RWC). Furthermore, proline (PRO) demonstrated a positive correlation with lipid peroxidation (MDA) and with the accessory pigments, chlorophyll b (Chlb), carotenoids (Car), and anthocyanins (Anth) (Fig. 11B).

## Discussion

After ten days of irrigation suspension, the plants showed a marked decrease in RWC, characterizing severe to moderate water stress, according to the physiological limits proposed by Laxa et al.^44^. The recovery of RWC after rehydration was consistent with observations in citrus and soybean^45,46^, indicating that cowpea maintains an intrinsic capacity to restore its water status. However, the magnitude of this recovery differed between treatments, revealing contrasting mechanisms promoted by Si and Met. Silicon favored greater restoration of RWC not only through structural effects, such as deposition in the cuticle and stomatal modulation^47^, but, above all, through functional mechanisms associated with efficiency in water use and redistribution, reflected in the osmotic adjustment mediated by TSS accumulation observed in this study. These responses indicate that Si increases the plant’s ability to maintain the water gradient and reduce transpiration fluxes under deficit, contributing to greater water stability during recovery. On the other hand, methionine promoted RWC recovery predominantly by stabilizing membranes, evidenced by the simultaneous reduction in EL and MDA. This response suggests that Met acts by reinforcing cellular integrity and limiting oxidative damage, preserving water retention in tissues, as described by Mehak et al.^48^. Thus, while Si acts mainly in the physical-functional control of water flow, Met acts in the biochemical protection of membranes—distinct mechanisms that converge towards greater water plasticity under stress and during rehydration.

The intensification of water deficit, reflected by the increase in MDA, confirms the progression of cellular dehydration and the loss of membrane integrity. Rehydration reduced this marker, but differently between stages and treatments. In the V3 stage, control plants maintained high MDA levels even after water restoration, evidencing greater vulnerability to oxidation. In contrast, at stage R1, the significant drop in MDA after the return of irrigation suggests a process of physiological memory, in which prior exposure to stress reinforces protective mechanisms activated in subsequent cycles^49^. Supplementation with Si potentiated this recovery, indicating that its effects are not limited to structural reinforcement, but also include the functional regulation of genes associated with water maintenance and osmotic balance, such as aquaporins^50^. The more pronounced decrease in MDA in plants treated with Si indicates that this element directly contributes to MDA restriction, favoring membrane stability and facilitating homeostatic re-establishment during rehydration.

Variations in TSP reinforce this integrated interpretation. At stage V3, water deficit significantly reduced TSP, reflecting the metabolic redirection typical of adverse conditions, in which protein degradation provides osmotically active amino acids to maintain cellular water potential^10^. Methionine, however, attenuated this decline by sustaining protein synthesis, reinforcing the increase in structural and enzymatic proteins, a mechanism already described for cowpea under stress^10^. This effect is not limited to Met’s role as a protein precursor, its action in activating antioxidant pathways, including phenolic compounds, ascorbic acid, and antioxidant enzymes, confers greater metabolic stability, reducing both protein degradation and the accumulation of oxidative damage^51^. Thus, while Si reduces membrane damage and favors the maintenance of water status through a physical-functional control of water, Met reinforces the plant’s biochemical capacity to suppress oxidative damage and preserve essential proteins. The combination of these mechanisms explains the greater plasticity of responses observed under stress and, especially, during water recovery.

The antioxidant complex showed distinct patterns between treatments, periods, and phenological stages, reflecting fine adjustments in response to variations in ROS generation. During water stress, the limitation of stomatal opening reduces photochemical dissipation and favors the accumulation of excess energy, resulting in the formation of superoxide radicals. SOD activation constitutes the first line of defense, converting ROS and reducing the risk of severe oxidative damage^52^. Subsequently, CAT and APX act in the removal of H₂O₂, but in distinct concentration ranges, which explains part of the contrasting responses observed between stages and treatments^53,54^. The predominance of CAT in the control treatment during stress suggests higher H₂O₂ concentrations, indicating a more intense oxidative overload compared to treatments with Si and Met. In contrast, the improvements observed in plants supplemented with Si and Met are associated with the strengthening of the antioxidant system, but through distinct physiological pathways. Silicon, by reducing the intensity of water stress and membrane damage, indirectly modulates ROS production, decreasing the demand on the antioxidant system. Methionine, in turn, acts directly as a metabolic regulator, inducing the synthesis of enzymatic and non-enzymatic antioxidants, reinforcing the cellular detoxification capacity. In the present study, this distinction was evident in the more expressive response of APX, whose action is favored under conditions of lower H₂O₂ concentration. This indicates that Si and Met, although distinct in their modes of action, converge towards a physiological state in which the accumulation of H₂O₂ is reduced to manageable levels, allowing APX to play a central role in maintaining redox homeostasis. Thus, the combination of functional attenuation of stress and antioxidant metabolic reinforcement explains the lower intensity of oxidative damage observed in treatments supplemented with these mitigators.

In addition to the antioxidant system, osmoprotection is an essential component of tolerance to water deficit, with PRO being one of the main osmolites involved. Although treatments with Si and Met did not promote significant differences in the present study, the strong influence of the evaluation periods confirms that PRO responds directly to the intensity of water stress, acting as a cellular stabilizer, as reported by Cavalcante et al. ^6^. Its accumulation contributes to the maintenance of leaf turgor, protection of membranes and preservation of cell volume, functions consistent with the responses observed under water restriction. The sharp drop after rehydration suggests rapid remobilization or degradation, a pattern already described in the literature and dependent on the severity and duration of stress^55,56^, reinforcing that its dynamics are highly plastic and adjusted to immediate physiological needs. Osmoprotection mediated by PRO is complemented by osmoregulation associated with soluble carbohydrates, whose dynamics were particularly evident during stress. The accumulation of sugars results from the reduction in translocation and consumption of photoassimilates, contributing to the maintenance of cellular osmotic potential^57^. Although this accumulation may temporarily reduce growth due to lower assimilate export, it ensures the minimum continuity of photosynthesis and preserves stomatal opening under low water potential. In the treatments with Si and Met, however, this osmotic accumulation assumed a dual character; in addition to contributing to osmotic adjustment during water deficit, these reserves were subsequently mobilized to support the resumption of biomass synthesis, which explains the observed increase in TDM. This behavior suggests that Si favors a more efficient distribution of water and preserves the integrity of the photosynthetic apparatus, while Met reinforces metabolic stability and the enzymatic capacity for remobilization. Thus, both treatments amplify the metabolic plasticity necessary for the transition between stress and recovery, albeit through distinct physiological pathways.

Silicon exerted consistent effects on the preservation of photosynthetic pigments, one of the most sensitive targets to water stress. The reduction of these pigments under water deficit results from limitations in water absorption and transport, compromising both pigment synthesis and chloroplast integrity^58^. The higher maintenance of these pigments in plants treated with Si suggests that the element acted not only as a physical barrier to water loss, but also as a modulator of oxidative status, as observed in studies demonstrating less chlorophyll degradation associated with the strengthening of the antioxidant system^59,60^. Thus, Si contributed to preserving photochemical efficiency during stress and accelerating its recovery after rehydration, reflecting an integrative effect between membrane stability, less ROS accumulation, and maintenance of photosynthetic structures. The growth dynamics reinforce this interpretation. Water stress significantly reduced leaf expansion and total dry mass accumulation, a typical response resulting from photosynthetic limitation, reduced turgor, and metabolic slowdown^6,61^. After rehydration, the restoration of cellular hydration allowed the gradual recovery of photosynthetic functions and carbon metabolism, promoting compensatory growth. In treatments with Si, this recovery was more efficient, resulting from a coordinated set of mechanisms, including greater oxidative stability, more effective osmotic adjustments, and preservation of pigments, which favored the restoration of photosynthesis and the subsequent accumulation of biomass.

Methionine also favored growth under stress and during recovery, but through mechanisms distinct from those observed for Si. As an essential amino acid, Met acts as a metabolic regulator and precursor of methylation reactions associated with the phenylpropanoid pathway, promoting the synthesis of lignin and other metabolites related to cellular stability and the mechanical resistance of tissues^62^. This structural action is complemented by the strengthening of the antioxidant system induced by Met, which reduces oxidative damage and maintains metabolic functionality under water deficit. Thus, while Si acts predominantly in maintaining the photosynthetic apparatus and water status, Met reinforces the biosynthesis of structural components and antioxidant capacity. Taken together, these results demonstrate that Si and Met enhance the physiological plasticity of cowpea through complementary pathways; Si directly protects and restores photosynthetic function, while Met strengthens structural stability and redox homeostasis. The convergence of these mechanisms explains the mitigation of the effects of water stress and the more efficient recovery of growth after rehydration.

The response of cowpea to water stress showed coordinated adjustments in leaf structure, photosynthetic pigments, and growth, modulated distinctly by Si and Met. The reduction in leaf area and leaching area reflected mesophyll impairment and a decrease in the functional area available for photosynthesis, while the decrease in leaching area indicated resource reallocation for structural maintenance and reduction of water losses. These patterns characterize a typical adaptive response of metabolic deceleration under limited water supply. Si attenuated these effects mainly through structural and physicochemical mechanisms. Its deposition in the epidermis reinforces cell walls and reduces vapor permeability, preserving leaf area and delaying leaf disorganization. This reinforcement extended to the photosynthetic apparatus; Si contributed to maintaining higher pigment levels, reducing pigment degradation induced by water limitation. This is associated with greater membrane stability and lower lipid peroxidation, indicating mitigation of oxidative damage and less ROS accumulation. Consequently, plants treated with Si better preserved photochemical efficiency and exhibited faster recovery after rehydration.

Met acted through metabolic and regulatory pathways, contrasting with the predominantly structural character of Si. As a precursor of signaling molecules and the phenylpropanoid pathway, Met can modulate mesophyll organization and favor the maintenance of functional leaf area, even with natural reductions in SLA during the reproductive phase. Furthermore, its action as an inducer of the antioxidant system contributes to reducing oxidative damage and preserving photosynthetic functionality, which sustains growth even under water deficit. In an integrated manner, the structural effects of Si and the metabolic adjustments of Met enhanced the physiological plasticity of the crop, resulting in less impairment of the active leaf area, greater stability of photosynthetic pigments, and better resumption of growth, reflecting a mechanistic complementarity between structural reinforcement and metabolic resilience.

The reduction in LAI in the control group reflected the loss of TLA and the photosynthetic limitation caused by the decrease in stomatal conductance, which compromised light interception and reduced primary productivity. This decline directly impacted NAR, indicating lower availability of photoassimilates to sustain growth during stress. The decrease in CGR reinforces this pattern, especially in the V3 phase, where higher metabolic demand makes it more sensitive to water deficit. However, the mitigation of these effects by Si and Met revealed contrasting mechanisms. Si acted mainly through structural and hydrological pathways, reducing transpiration losses, preserving RWC, and sustaining cell elongation and expansion. This preservation of hydration favored higher values of LAI, NAR, and CGR, reflecting the maintenance of net assimilation even under water restriction. Met, on the other hand, acted metabolically and regulatory, intensifying components of the antioxidant system and TSP content, which reduced lipid peroxidation damage and preserved photosynthetic functionality. With less oxidative damage, the leaves remained active for longer, which was reflected in higher LAD values at the R1 stage, in contrast to the sharp decline observed in the control. Thus, although both elicitors favored photosynthetic recovery and biomass accumulation after rehydration, they did so through different pathways: Si favored greater water retention and preservation of cell expansion, while Met contributed to metabolic and antioxidant strengthening, reducing damage and prolonging leaf functionality. This complementarity shows that Si and Met enhance the physiological plasticity of cowpea by modulating different levels of control, resulting in greater growth resilience under water stress. Water use efficiency is directly related to net photosynthesis, stomatal conductance, transpiration, and leaf water status, all severely affected by water deficit and responsible for biomass reduction in cowpea. Under stress conditions, stomatal conductance tends to decrease more than assimilation, potentially momentarily increasing WUE, a pattern that was accentuated in plants treated with Si, whose leaf deposits reduced water loss, improved stomatal regulation, and reduced transpiration, allowing for more efficient conversion of transpired water into Total Dry Mass.

Correlation analysis indicated two functional clusters: one associated with the maintenance of photosynthesis and growth, and another linked to the activation of antioxidant and osmotic defense mechanisms, whose intensity and direction varied according to the elicitor. In plants with Si, strong positive correlations prevailed between RWC, pigments, and growth variables, suggesting that Si preserves water integrity and photosynthetic apparatus, reducing MDA and minimizing the need for costly antioxidant responses. In plants with Met, although there is also a positive association between growth and pigments, more negative correlations emerged between growth markers and defense indicators, indicating that Met induces strong biochemical activation to control ROS, increasing TSP and enzymatic activities, but at a higher physiological cost by diverting carbon and energy to defense; even so, this response was sufficient to reduce oxidative damage and improve performance under stress. In summary, Si favors WUE and TDM accumulation mainly through physical-structural and water preservation pathways, while Met operates via metabolic-antioxidant reinforcement, prolonging LAD and leaf functionality in critical stages, which explains the complementarity observed in water deficit mitigation strategies.

In general, silicon showed more integrated effects on growth, expressed by positive correlations between photosynthetic pigments, total soluble proteins, and antioxidant system variables, indicating that the preservation of photosynthetic integrity, combined with higher Relative Water Content and reduced lipid peroxidation, supported biomass accumulation under water deficit. Methionine, in turn, also promoted favorable coupling between antioxidant enzymes and TSP, but with patterns that suggest a higher physiological cost, since the increase in enzymes and soluble proteins reflected strong metabolic activation for ROS control. Even so, this biochemical response was sufficient to improve plant performance, aligning with the antioxidant coordination model described by Alencar et al.^64^, Shi et al.^65^ e Kankia et al.^66^. In both elicitors, growth gains were strongly associated with the maintenance of functional leaf area and greater Leaf Area Duration, confirming the importance of these attributes for sustaining photosynthesis and, consequently, growth, as highlighted by Cavalcante et al.^6^.

In summary, Si and Met induce distinct, yet complementary, physiological responses. Si acts mainly through physical-structural and hydrological mechanisms, preserving RWC, photosynthetic apparatus, and water use efficiency. Met operates predominantly via metabolic-antioxidant reinforcement to limit oxidative damage and maintain leaf functionality. Despite advances, relevant gaps remain, including the evaluation of these elicitors in different cultivars, concentrations, and application methods, as well as validations under field conditions to determine their agronomic viability. Future studies addressing these dimensions will be fundamental to consolidating the use of Si and Met as effective strategies for mitigating water stress in agricultural systems.

## Conclusion

In conclusion, cowpea plants suffered water stress during the drought period, evidenced by reduced water status, increased indicators of membrane damage, and greater mobilization of osmoregulation mechanisms and antioxidant complexes. In addition, growth was reduced, reflected in decreased dry matter and leaf area, as well as biomass allocation for uses unrelated to growth. Elicitors improved some plant attributes, favoring development even under stress, and demonstrated faster recovery. Silicon was the element most closely related to the growth apparatus, according to the results, although it also reflected improvements in biochemical metabolism. Methionine, on the other hand, is associated with changes in plant physiology, particularly in biochemical aspects, which directly impact growth. In both cases, the improvements observed during the drought were those that enabled greater recovery.

## Supplementary Information

Below is the link to the electronic supplementary material.

Supplementary Material 1

## Data Availability

The data will be available upon request to the corresponding author, Alberto Soares de Melo (e-mail: [alberto.melo@servidor.uepb.edu.br](mailto: alberto.melo@servidor.uepb.edu.br) ).
