# Supplementary material for: Silicon and methionine enhance cowpea water stress tolerance
Source: Sci Rep. 2026 Jan 31;16:6800. doi: 10.1038/s41598-026-37795-2 (PMC12917182; doi:10.1038/s41598-026-37795-2)
Supplement: Supplementary file 1 — Supplementary Material 1 [file 41598_2026_37795_MOESM1_ESM.pdf]

**Supplementary materials**

**Table 1.** Physicochemical characteristics of the soil used in the experiment.

| Physical characteristics | Chemical characteristics |
|---|---|
| Sand (89.64%) | Nitrogen (0.88 g $kg^{-1}$) |
| Silt (10.04%) | Phosphorus (658.66 mg $kg^{-1}$) |
| Clay (0.32%) | Potassium (0.97 cmolc $kg^{-1}$) |
| Soil density (1.32 g $cm^{-3}$) | Calcium (4.41 cmolc $kg^{-1}$) |
| Particle density (2.73 g $cm^{-3}$) | Magnesium (4.75 cmolc $kg^{-1}$) |
| Porosity (52.38%) | Sodium (0.04 cmolc $kg^{-1}$) |
| | Sulfur (10.17 cmolc $kg^{-1}$) |
| | Organic matter (30.46 g $kg^{-1}$) |
| | Organic carbon (17.67 g $kg^{-1}$) |
| | CEC (10.17 cmolc $kg^{-1}$) |
| | Hydrogen (0.00 cmolc $kg^{-1}$) |
| | Aluminum (0.00 cmolc $kg^{-1}$) |
| | pH (7.02) |
| | Electrical conductivity (0.83 mmhos $cm^{-1}$) |
| | Available water (3.43% dry soil basis) |

**Table 2.** Means, standard errors, and effect sizes of physiological, biochemical, and growth-related variables in V3 stage cowpea plants under water restriction and rehydration.

| RWC (%) | | | EL (%) | | |
|---|---|---|---|---|---|
| | With restriction | After rehydration | | With restriction | After rehydration |
| Control | 57.24 ± 0.70 | 68.65 ± 0.40 | Control | 55.78 ± 2.10 | 45.23 ± 1.68 |
| | Effect Size: -6.269 | | | Effect Size: 1.790 | |
| Si | 58.15 ± 0.58 | 76.76 ± 1.08 | Si | 41.78 ± 9.70 | 41.42 ± 0.82 |
| | Effect Size: -8.277 | | | Effect Size:0.018 | |
| Met | 62.18 ± 1.37 | 73.26 ± 2.49 | Met | 37.50 ± 4.16 | 38.09 ± 1.94 |
| | Effect Size: -2.106 | | | Effect Size: -0.058 | |
| Si + Met | 60.08 ± 1.04 | 70.07 ± 0.30 | Si + Met | 40.95 ± 10.93 | 40.95 ± 0.67 |
| | Effect Size: -4.175 | | | Effect Size: 0.150 | |
| nmol MDA $g^{-1}$ FM | | | mg TSPs $g^{-1}$ FM | | |
| | With restriction | After rehydration | | With restriction | After rehydration |
| Control | 6.97 ± 0.14 | 5.29 ± 0.11 | Control | 1.55 ± 0.21 | 4.05 ± 0.33 |
| | Effect Size: 4.168 | | | Effect Size: -3.256 | |
| Si | 3.56 ± 0.95 | 2.84 ± 0.05 | Si | 3.83 ± 0.59 | 4.08 ± 0.23 |
| | Effect Size: 0.369 | | | Effect Size: -0.175 | |
| Met | 2.64 ± 0.44 | 1.09 ± 0.02 | Met | 6.00 ± 1.42 | 4.75 ± 0.72 |
| | Effect Size: 1.699 | | | Effect Size: 0.349 | |
| Si + Met | 1.72 ± 0.09 | 4.42 ± 0.14 | Si + Met | 5.72 ± 0.33 | 2.14 ±0.42 |
| | Effect Size: -8.059 | | | Effect Size: 3.249 | |
| UA SOD $g^{-1}$ FM | | | nM $H_2O_2$ $min^{-1}$ $g^{-1}$ protein | | |
| | With restriction | After rehydration | | With restriction | After rehydration |
| Control | 89.57 ± 4.74 | 71.90 ± 9.52 | Control | 16.72 ± 2.44 | 6.10 ± 0.70 |
| | Effect Size: 0.929 | | | Effect Size: 1.899 | |
| Si | 75.95 ± 4.92 | 84.12 ± 18.65 | Si | 8.86 ± 1.61 | 7.64 ± 1.41 |
| | Effect Size: -0.287 | | | Effect Size: 0.264 | |
| Met | 99.04 ± 10.85 | 71.58 ± 14.02 | Met | 4.89 ± 1.67 | 4.13 ± 1.04 |
| | Effect Size: 0.769 | | | Effect Size: 0.172 | |
| Si + Met | 88.69 ± 10.89 | 64.28 ± 14.58 | Si + Met | 3.46 ± 0.62 | 5.33 ± 1.02 |
| | Effect Size: 0.671 | | | Effect Size: -0.861 | |

| nM ascorbate $min^{-1}$ $g^{-1}$ protein | With restriction | After rehydration |
|---|---|---|
| Control | 74.78 ± 9.36 | 22.12 ± 2.41 |
| | Effect Size: 2.491 | |
| Si | 42.01 ± 5.61 | 34.77 ± 7.15 |
| | Effect Size: 0.394 | |
| Met | 37.57 ± 11.64 | 22.17 ± 8.16 |
| | Effect Size: 0.490 | |
| Si + Met | 40.01 ± 14.56 | 85.45 ± 1.78 |
| | Effect Size: -1.468 | |

| μmol PRO $g^{-1}$ FM | With restriction | After rehydration |
|---|---|---|
| Control | 2.28 ± 0.53 | 0.37 ± 0.03 |
| | Effect Size: 1.703 | |
| Si | 1.90 ± 0.19 | 0.69 ± 0.16 |
| | Effect Size: 2.214 | |
| Met | 2.82 ± 0.85 | 0.55 ± 0.16 |
| | Effect Size: 1.209 | |
| Si + Met | 2.82 ± 0.18 | 0.97 ± 0.53 |
| | Effect Size: 2.052 | |

| mg TSS $g^{-1}$ FM | With restriction | After rehydration |
|---|---|---|
| Control | 11.82 ± 0.85 | 6.47 ± 0.24 |
| | Effect Size: 2.704 | |
| Si | 10.10 ± 0.52 | 6.17 ± 1.49 |
| | Effect Size: 1.540 | |
| Met | 8.66 ± 1.75 | 7.61 ± 0.42 |
| | Effect Size: 0.267 | |
| Si + Met | 8.10 ± 0.35 | 9.38 ± 0.79 |
| | Effect Size: -0.850 | |

| mg SUC $g^{-1}$ FM | With restriction | After rehydration |
|---|---|---|
| Control | 3.50 ± 0.21 | 3.45 ± 0.21 |
| | Effect Size: 0.079 | |
| Si | 3.33 ± 0.12 | 2.69 ± 0.26 |
| | Effect Size: 1.219 | |
| Met | 3.53 ± 0.70 | 3.4 ± 0.13 |
| | Effect Size: 0.088 | |
| Si + Met | 3.18 ± 0.04 | 4.30 ± 0.05 |
| | Effect Size: -7.506 | |

| μg Chla 100 $g^{-1}$ FM | With restriction | After rehydration |
|---|---|---|
| Control | 1.13 ± 0.06 | 1.02 ± 0.07 |
| | Effect Size: 0.511 | |
| Si | 3.11 ± 0.70 | 1.05 ± 0.00 |
| | Effect Size: 1.442 | |
| Met | 1.75 ± 0.49 | 1.05 ± 0.01 |
| | Effect Size: 0.696 | |
| Si + Met | 1.20 ± 0.03 | 1.12 ± 0.00 |
| | Effect Size: 1.227 | |

| μg Chlb 100 $g^{-1}$ FM | With restriction | After rehydration |
|---|---|---|
| Control | 1.40 ± 0.07 | 1.60 ± 0.05 |
| | Effect Size: -1.043 | |
| Si | 3.90 ± 1.55 | 1.85 ± 0.06 |
| | Effect Size: 0.647 | |
| Met | 2.58 ± 1.00 | 1.78 ± 0.01 |
| | Effect Size: 0.398 | |
| Si + Met | 1.77 ± 0.24 | 1.68 ± 0.04 |
| | Effect Size: 0.182 | |

| μg Chl total 100 $g^{-1}$ FM | With restriction | After rehydration |
|---|---|---|
| Control | 2.50 ± 0.05 | 2.62 ± 0.12 |
| | Effect Size: -0.535 | |
| Si | 7.89 ± 1.99 | 2.81 ± 0.02 |
| | Effect Size: 1.265 | |
| Met | 4.78 ± 1.39 | 2.82 ± 0.02 |
| | Effect Size: 0.694 | |
| Si + Met | 3.22 ± 0.06 | 2.77 ± 0.02 |
| | Effect Size: 3.032 | |

| μg Car 100 $g^{-1}$ FM | With restriction | After rehydration |
|---|---|---|
| Control | 5.01 ± 0.33 | 5.07 ± 0.84 |
| | Effect Size: -0.041 | |
| Si | 6.18 ± 1.51 | 4.50 ± 0.32 |
| | Effect Size: 0.503 | |
| Met | 5.57 ± 1.38 | 4.30 ± 0.38 |
| | Effect Size: 0.402 | |
| Si + Met | 4.81 ± 0.88 | 4.04 ± 0.39 |
| | Effect Size: 0.354 | |

| µg Anth 100 g⁻¹ FM | With restriction | After rehydration | Effect Size |
|---|---|---|---|
| Control | 8.68 ± 0.21 | 8.90 ± 0.43 | -0.254 |
| Si | 21.72 ± 7.11 | 9.39 ± 0.45 | 0.839 |
| Met | 10.09 ± 1.64 | 9.41 ± 0.35 | 0.186 |
| Si + Met | 9.02 ± 1.28 | 9.13 ± 0.19 | -0.040 |

| TLA ($cm^2$) | With restriction | After rehydration | Effect Size |
|---|---|---|---|
| Control | 129.77 ± 14.02 | 232.5 ± 21.60 | -2.068 |
| Si | 175.09 ± 3.04 | 260.41 ± 47.33 | -1.597 |
| Met | 185.13 ± 5.16 | 253.85 ± 18.84 | -2.356 |
| Si + Met | 180.43 ± 12.86 | 237.44 ± 10.26 | -1.584 |

| SLA ($mm^2$ $mg^{-1}$) | With restriction | After rehydration | Effect Size |
|---|---|---|---|
| Control | 29.30 ± 1.47 | 27.35 ± 2.04 | 0.309 |
| Si | 31.31 ± 2.97 | 33.16 ± 2.79 | -0.213 |
| Met | 37.13 ± 1.49 | 33.66 ± 3.32 | 0.550 |
| Si + Met | 32.44 ± 2.07 | 27.41 ± 0.44 | 1.097 |

| TDM (g) | With restriction | After rehydration | Effect Size |
|---|---|---|---|
| Control | 1.45 ± 0.08 | 1.71 ± 0.11 | 1.899 |
| Si | 1.34 ± 0.05 | 2.36 ± 0.19 | 0.264 |
| Met | 1.53 ± 0.06 | 2.07 ± 0.10 | 0.172 |
| Si + Met | 1.51 ± 0.08 | 1.96 ± 0.10 | -0.861 |

| LAR ($cm^2$ $mg^{-1}$) | With restriction | After rehydration | Effect Size |
|---|---|---|---|
| Control | 0.08 ± 0.01 | 0.13 ± 0.01 | -1.462 |
| Si | 0.13 ± 0.00 | 0.11 ± 0.02 | 0.407 |
| Met | 0.12 ± 0.00 | 0.12 ± 0.01 | -0.094 |
| Si + Met | 0.11 ± 0.00 | 0.12 ± 0.01 | -0.095 |

| LMR ($g$ $g^{-1}$) | With restriction | After rehydration | Effect Size |
|---|---|---|---|
| Control | 0.43 ± 0.05 | 0.54 ± 0.00 | -0.958 |
| Si | 0.44 ± 0.00 | 0,48 ± 0.01 | -1.268 |
| Met | 0.48 ± 0.00 | 0.50 ± 0.00 | -0.656 |
| Si + Met | 0.48 ± 0.02 | 0.51 ± 0.00 | -0.516 |

| LAI ($cm^2$ $cm^{-2}$) | With restriction | After rehydration | Effect Size |
|---|---|---|---|
| Control | 0.45 ± 0.04 | 0.82 ± 0.07 | -2.068 |
| Si | 0.61 ± 0.01 | 0.91 ± 0.16 | -1.597 |
| Met | 0.65 ± 0.01 | 0.89 ± 0.06 | -2.356 |
| Si + Met | 0.63 ± 0.04 | 0.83 ± 0.03 | -1.584 |

*Values are presented as mean ± standard error. The evaluated variables were total soluble protein (TSP), superoxide dismutase (SOD), catalase (CAT), ascorbate peroxidase (APX), proline (PRO), total soluble sugars (TSS), sucrose (SUC), lipid peroxidation (MDA), chlorophyll *a* (Chl *a*), chlorophyll *b* (Chl *b*), carotenoids (Car), anthocyanins (Anth), total chlorophyll (Chl total), relative water content (RWC, %), electrolyte leakage (EL), total dry mass (TDM), total leaf area (TLA), specific leaf area (SLA), leaf area ratio (LAR), leaf mass ratio (LMR), and leaf area index (LAI). Cowpea plants ("BRS Exuberante") at the V3 stage were treated with silicon (Si), methionine (Met), or their combination (Si + Met) under water restriction and after rehydration. Effect size values indicate the magnitude of the difference between water restriction and rehydration conditions for each treatment.

**Table 3.** Means, standard errors, and effect sizes of physiological, biochemical, and growth-related variables in R1 stage cowpea plants under water restriction and rehydration.

| RWC (%) | | | EL (%) | | |
|---|---|---|---|---|---|
| | With restriction | After rehydration | | With restriction | After rehydration |
| Control | 71.83 ± 7.76 | 71.35 ± 4.84 | Control | 48.33 ± 10.67 | 45.00 ± 2.04 |
| | Effect Size: 0.024 | | | Effect Size: 0.143 | |
| Si | 79.65 ± 9.09 | 78.15 ± 5.30 | Si | 59.16 ± 8.85 | 36.83 ± 1.36 |
| | Effect Size: 0.064 | | | Effect Size: 1.170 | |
| Met | 71.41 ± 4.84 | 84.43 ± 5.73 | Met | 46.83 ± 5.44 | 32.5 ± 7.5 |
| | Effect Size: -0.844 | | | Effect Size: 0.779 | |
| Si + Met | 57.03 ± 2.45 | 85.91 ± 2.45 | Si + Met | 50.11 ± 5.97 | 32.25 ± 4.40 |
| | Effect Size: -3.576 | | | Effect Size: 1.092 | |
| nmol MDA g$^{-1}$ FM | | | mg TSPs g$^{-1}$ FM | | |
| | With restriction | After rehydration | | With restriction | After rehydration |
| Control | 6.29 ± 0.77 | 2.33 ± 0.10 | Control | 22.11 ± 2.76 | 21.14 ± 2.59 |
| | Effect Size: 2.393 | | | Effect Size: 0.119 | |
| Si | 1.66 ± 0.07 | 0.68 ± 0.02 | Si | 18.50 ± 1.91 | 16.59 ± 3.46 |
| | Effect Size: 5.722 | | | Effect Size: 0.262 | |
| Met | 2.05 ± 0.19 | 0.56 ± 0.05 | Met | 24.88 ± 2.08 | 22.70 ± 2.04 |
| | Effect Size: 3.454 | | | Effect Size: 0.351 | |
| Si + Met | 1.26 ± 0.05 | 0.62 ± 0.02 | Si + Met | 24.72 ± 0.26 | 18.2 ± 1.17 |
| | Effect Size: 4.740 | | | Effect Size: 3.810 | |
| UA SOD g$^{-1}$ FM | | | nM $H_2O_2$ min$^{-1}$ g$^{-1}$ protein | | |
| | With restriction | After rehydration | | With restriction | After rehydration |
| Control | 90.35 ± 5.72 | 81.19 ± 4.17 | Control | 0.40 ± 0.06 | 0.74 ± 0.10 |
| | Effect Size: | | | Effect Size: -1.483 | |
| Si | 98.33 ± 4.35 | 64.40 ± 3.80 | Si | 0.37 ± 0.04 | 0.85 ± 0.32 |
| | Effect Size: | | | Effect Size: -1.473 | |
| Met | 94.88 ± 7.28 | 75.35 ± 4.52 | Met | 0.37 ± 0.00 | 0.40 ± 0.04 |
| | Effect Size: | | | Effect Size: -0.574 | |
| Si + Met | 98.33 ± 0.09 | 95.87 ± 1.17 | Si + Met | 0.34 ± 0.06 | 0.72 ± 0.12 |
| | Effect Size: | | | Effect Size: -1.459 | |
| nM ascorbate min$^{-1}$ g$^{-1}$ protein | | | µmol PRO g$^{-1}$ FM | | |
| | With restriction | After rehydration | | With restriction | After rehydration |
| Control | 3.55 ± 0.64 | 2.59 ± 0.31 | Control | 4.45 ± 1.29 | 0.28 ± 0.14 |
| | Effect Size: 0.599 | | | Effect Size: 1.523 | |
| Si | 6.22 ± 0.80 | 8.00 ± 2.14 | Si | 2.25 ± 0.38 | 0.33 ± 0.03 |
| | Effect Size: -0.472 | | | Effect Size: 2.207 | |
| Met | 3.22 ± 0.24 | 6.78 ± 1.20 | Met | 3.07 ± 0.68 | 0.20 ± 0.04 |
| | Effect Size: -2.096 | | | Effect Size: 2.031 | |
| Si + Met | 8.56 ± 0.49 | 8.16 ± 0.80 | Si + Met | 4.18 ± 0.04 | 0.33 ± 0.04 |
| | Effect Size: 0.221 | | | Effect Size: 6.264 | |

| mg TSS $g^{-1}$ FM | With restriction | After rehydration | Effect Size |
|---|---|---|---|
| Control | 18.51 ± 2.63 | 9.31 ± 0.90 | 1.493 |
| Si | 15.05 ± 1.74 | 9.85 ± 1.08 | 1.138 |
| Met | 13.26 ± 1.73 | 11.13 ± 0.82 | 0.495 |
| Si + Met | 10.65 ± 0.58 | 8.93 ± 0.15 | 1.293 |

| mg SUC $g^{-1}$ FM | With restriction | After rehydration | Effect Size |
|---|---|---|---|
| Control | 9.08 ± 1.03 | 6.07 ± 0.28 | 1.280 |
| Si | 6.20 ± 0.61 | 5.09 ± 0.65 | 0.581 |
| Met | 6.28 ± 0.56 | 6.68 ± 0.56 | -0.277 |
| Si + Met | 7.79 ± 0.60 | 6.36 ± 0.24 | 0.973 |

| µg Chla 100 $g^{-1}$ FM | With restriction | After rehydration | Effect Size |
|---|---|---|---|
| Control | 32.12 ± 0.70 | 29.28 ± 3.45 | 0.584 |
| Si | 31.79 ± 0.35 | 32.47 ± 0.48 | -0.565 |
| Met | 31.82 ± 0.41 | 28.39 ± 4.79 | 0.610 |
| Si + Met | 33.21 ± 0.24 | 33.38 ± 0.24 | -0.198 |

| µg Chlb 100 $g^{-1}$ FM | With restriction | After rehydration | Effect Size |
|---|---|---|---|
| Control | 18.91 ± 0.98 | 22.42 ± 7.22 | -0.382 |
| Si | 27.85 ± 4.40 | 26.66 ± 5.90 | 0.081 |
| Met | 33.36 ± 4.65 | 22.35 ± 6.51 | 0.696 |
| Si + Met | 32.16 ± 1.30 | 27.84 ± 2.05 | 0.926 |

| µg Chlt 100 $g^{-1}$ FM | With restriction | After rehydration | Effect Size |
|---|---|---|---|
| Control | 51.04 ± 1.60 | 51.71 ± 9.60 | -0.052 |
| Si | 59.65 ± 4.75 | 59.13 ± 5.50 | 0.035 |
| Met | 65.19 ± 4.23 | 50.74 ± 10.38 | 0.766 |
| Si + Met | 65.38 ± 1.40 | 61.22 ± 1.69 | 0.924 |

| µg Car 100 $g^{-1}$ FM | With restriction | After rehydration | Effect Size |
|---|---|---|---|
| Control | 14.12 ± 0.54 | 12.00 ± 1.09 | 0.973 |
| Si | 14.72 ± 1.58 | 12.90 ± 0.88 | 0.450 |
| Met | 12.67 ± 1.10 | 13.30 ± 0.79 | -0.213 |
| Si + Met | 13.06 ± 0.60 | 13.29 ± 0.40 | -0.144 |

| µg Anth 100 $g^{-1}$ FM | With restriction | After rehydration | Effect Size |
|---|---|---|---|
| Control | 32.27 ± 3.24 | 33.17 ± 8.77 | -0.059 |
| Si | 31.88 ± 7.78 | 38.19 ± 6.46 | -0.286 |
| Met | 38.95 ± 7.55 | 32.02 ± 8.23 | 0.297 |
| Si + Met | 39.34 ± 3.18 | 38.70 ± 2.10 | 0.075 |

| TLA ($cm^2$) | With restriction | After rehydration | Effect Size |
|---|---|---|---|
| Control | 561.89 ± 12.41 | 631.11 ± 7.56 | -2.137 |
| Si | 725.316 ± 55.66 | 745.31 ± 58.54 | -0.121 |
| Met | 600.89 ± 8.21 | 668.61 ± 12.17 | -2.367 |
| Si + Met | 549.56 ± 26.58 | 578.41 ± 21.41 | -0.387 |

| | SLA ($mm^2$ $mg^{-1}$) | | TDM (g) | |
|---|---|---|---|---|
| | With restriction | After rehydration | With restriction | After rehydration |
| Control | 36.58 ± 3.10 | 29.93 ± 3.73 | 3.62 ± 0.15 | 5.02 ± 0.31 |
| | Effect Size: -0.059 | | Effect Size: -2.146 | |
| Si | 31.60 ± 3.79 | 30.29 ± 1.67 | 4.78 ± 0.28 | 5.93 ± 0.39 |
| | Effect Size: -0.286 | | Effect Size: -1.197 | |
| Met | 32.27 ± 3.43 | 24.89 ± 0.87 | 4.14 ± 0.22 | 5.39 ± 0.15 |
| | Effect Size: 0.297 | | Effect Size: -2.082 | |
| Si + Met | 24.78 ± 0.94 | 28.33 ± 1.96 | 4.80 ± 0.34 | 6.03 ± 0.08 |
| | Effect Size: 0.075 | | Effect Size: -1.610 | |

| | LAR ($cm^2$ $mg^{-1}$) | | LMR (g $g^{-1}$) | |
|---|---|---|---|---|
| | With restriction | After rehydration | With restriction | After rehydration |
| Control | 0.15 ± 0.00 | 0.12 ± 0.00 | 0.37 ± 0.01 | 0.47 ± 0.02 |
| | Effect Size: 1.272 | | Effect Size: -2.224 | |
| Si | 0.15 ± 0.00 | 0.12 ± 0.01 | 0.40 ± 0.03 | 0.44 ± 0.02 |
| | Effect Size: 0.751 | | Effect Size: -0.362 | |
| Met | 0.14 ± 0.00 | 0.12 ± 0.00 | 0.41 ± 0.02 | 0.45 ± 0.01 |
| | Effect Size: 1.067 | | Effect Size: -0.614 | |
| Si + Met | 0.11 ± 0.00 | 0.09 ± 0.00 | 0.35 ± 0.01 | 0.44 ± 0.00 |
| | Effect Size: 1.137 | | Effect Size: -2.246 | |

| | LAI ($cm^2$ $cm^{-2}$) | |
|---|---|---|
| | With restriction | After rehydration |
| Control | 1.98 ± 0.04 | 2.22 ± 0.02 |
| | Effect Size: -2.137 | |
| Si | 2.55 ± 0.19 | 2.63 ± 0.02 |
| | Effect Size: -0.121 | |
| Met | 2.11 ± 0.02 | 2.35 ± 0.04 |
| | Effect Size: -2.367 | |
| Si + Met | 1.93 ± 0.09 | 2.04 ± 0.07 |
| | Effect Size: -0.387 | |

*Values are presented as mean ± standard error. The evaluated variables were total soluble protein (TSP), superoxide dismutase (SOD), catalase (CAT), ascorbate peroxidase (APX), proline (PRO), total soluble sugars (TSS), sucrose (SUC), lipid peroxidation (MDA), chlorophyll *a* (Chl *a*), chlorophyll *b* (Chl *b*), carotenoids (Car), anthocyanins (Anth), total chlorophyll (Chl total), relative water content (RWC, %), electrolyte leakage (EL), total dry mass (TDM), total leaf area (TLA), specific leaf area (SLA), leaf area ratio (LAR), leaf mass ratio (LMR), and leaf area index (LAI). Cowpea plants ("BRS Exuberante") at the R1 stage were treated with silicon (Si), methionine (Met), or their combination (Si + Met) under water restriction and after rehydration. Effect size values indicate the magnitude of the difference between water restriction and rehydration conditions for each treatment.

**Table 4.** Growth analysis parameters of V3 stage cowpea plants under silicon and methionine treatments.

| CGR (mg cm$^{-2}$ dia$^{-1}$) | | LAD (m² dia$^{-1}$) | |
|---|---|---|---|
| Control | 0.44 ± 0.05 | Control | 0.03 ± 0.00 |
| Si | 1.79 ± 0.24 | Si | 0.04 ± 0.00 |
| Met | 0.96 ± 0.21 | Met | 0.04 ± 0.00 |
| Si + Met | 0.79 ± 0.19 | Si + Met | 0.04 ± 0.00 |
| **NAR (mg cm$^{-2}$ dia$^{-1}$)** | | **WUE (g mm$^{-1}$)** | |
| Control | 0.73 ± 0.07 | Control | 1.63 ± 0.09 |
| Si | 2.49 ± 0.49 | Si | 2.24 ± 0.12 |
| Met | 1.29 ± 0.31 | Met | 1.95 ± 0.10 |
| Si + Met | 1.12 ± 0.31 | Si + Met | 2.00 ± 0.07 |

Values are presented as mean ± standard error. Net assimilatory rate (NAR), crop growth rate (CGR), leaf area duration (LAD), and water use efficiency (WUE) were evaluated in cowpea plants (cv. BRS Exuberante) at the V3 stage treated with silicon (Si), methionine (Met), or their combination (Si + Met). CGR and NAR are expressed as mg cm$^{-2}$ day$^{-1}$, LAD as m² day$^{-1}$, and WUE as g mm$^{-1}$.

**Table 5.** Growth analysis parameters of R1 stage cowpea plants under silicon and methionine treatments.

| CGR (mg cm$^{-2}$ dia$^{-1}$) | | LAD (m² dia$^{-1}$) | |
|---|---|---|---|
| Control | 2.46 ± 0.56 | Control | 0.11 ± 0.00 |
| Si | 2.02 ± 0.65 | Si | 0.14 ± 0.01 |
| Met | 2.19 ± 0.65 | Met | 0.12 ± 0.00 |
| Si + Met | 2.16 ± 0.67 | Si + Met | 0.11 ± 0.00 |
| **NAR (mg cm-2 dia-1)** | | **WUE (g mm-1)** | |
| Control | 1.18 ± 0.28 | Control | 2.24 ± 0.12 |
| Si | 0.84 ± 0.32 | Si | 2.73 ± 0.12 |
| Met | 0.97 ± 0.29 | Met | 2.34 ± 0.05 |
| Si + Met | 1.11 ± 0.34 | Si + Met | 2.87 ± 0.13 |

Values are presented as mean ± standard error. Net assimilatory rate (NAR), crop growth rate (CGR), leaf area duration (LAD), and water use efficiency (WUE) were evaluated in cowpea plants (cv. BRS Exuberante) at the R1 stage treated with silicon (Si), methionine (Met), or their combination (Si + Met). CGR and NAR are expressed as mg cm$^{-2}$ day$^{-1}$, LAD as m² day$^{-1}$, and WUE as g mm$^{-1}$.
